# Palladium and Platinum 2,4-*cis*-amino Azetidine and Related Complexes

**DOI:** 10.3389/fchem.2018.00211

**Published:** 2018-06-21

**Authors:** Akina Yoshizawa, Antonio Feula, Andrew G. Leach, Louise Male, John S. Fossey

**Affiliations:** ^1^School of Chemistry, University of Birmingham, Birmingham, United Kingdom; ^2^School of Pharmacy and Biomolecular Sciences, Liverpool John Moores University, Liverpool, United Kingdom; ^3^X-Ray Crystallography Facility, School of Chemistry, University of Birmingham, Birmingham, United Kingdom

**Keywords:** azetidine, ligand, platinum, palladium, complex, pyrrolidine

## Abstract

Seven *N,N*'-palladium(II) chloride complexes, one *N,N*'-palladium(II) acetate complex of 2,4-*cis*-azetidines where prepared and analyzed by single crystal XRD. Two platinum(II) chloride *N,N*'-complexes of 2,4-*cis*-azetidines where prepared and analyzed by single crystal XRD. Computational analysis and determination of the %Vbur was examined conducted. A *CNN*' metallocyclic complex was prepared by oxidative addition of palladium(0) to an ortho bromo 2,4-*cis*-disubstituted azetidine and its crystal structure displays a slightly pyramidalized metal-ligand orientation.

## Introduction

Complexes of group 10 metals coordinated by *N,N'*-ligands have found wide use from therapeutics (Graham et al., 2004) through to catalysis (Fossey et al., 2005; Saito and Fu, 2007; Binder et al., 2012; Martínez-Olid et al., 2014; Álvarez-Casao et al., 2015). In our research, through studying the synthesis and cyclisation of homoallylamine derivatives (Fossey et al.; Rixe et al., 1996; Jamieson and Lippard, 1999; Feula et al., 2010, 2013; Feula and Fossey, 2013; Hama Salih et al., 2015), the potential for use of the *cis*-aminoazetidine products as ligands emerged, and their use in asymmetric copper-catalyzed Henry reactions was developed and reported elsewhere (Yoshizawa et al., 2018a,b,c). Group 10 complexes of azetidine derivatives have been reported previously (Voureka et al., 1996; Keller et al., 2005; Lee et al., 2008, 2009a,b; Choi et al., 2011), and during our aforementioned studies various attempts to form and isolate racemic group 10 metal complexes have been made. Successful attempts to prepare crystals of metal complexes suitable for single crystal X-ray diffraction structure determination are reported herein, along with crystal structures of unexpected by-products, a complex formed from a pyrrolidine ligand (an isomer of the azetidines of primary interest) as well as an observation pertaining to diastereoisomer differentiation upon complexation and sample preparation under slightly different conditions. In most cases small amounts of (or even just single) crystals were obtained sometimes precluding full characterisation, however in some cases elemental analyses are reported. The structural observations facilitated by surveying the obtained crystal structures have the potential to influence catalysis design. Thus, whilst this report primarily serves as a crystal structure report the interpretation and understanding garnered is gathered here with a view to informing studies across organic and inorganic synthesis.

## Results and discussion

Our previously documented interest in the synthesis of 2,4-*cis*-disubstituted azetidines (Fossey et al.; Feula et al., 2010, 2013; Feula and Fossey, 2013; Hama Salih et al., 2015), and our recent use of the same family of azetidines as chiral ligands in asymmetric copper-catalyzed Henry reactions afforded us the opportunity to probe the complexation of these ligands and related compounds with various metal sources, in this report examples leading to samples suitable for analysis by X-ray single crystal diffraction studies are collected and compared. A previously revealed platinum complex (**4a**) is re-reported here since it is compared directly with a diastereomeric complex (**4b**) of the same ligand, and the racemic ligands used in this study have all been previously reported in the aforementioned publications arising from this programme of research^1^.

**Scheme 1:**
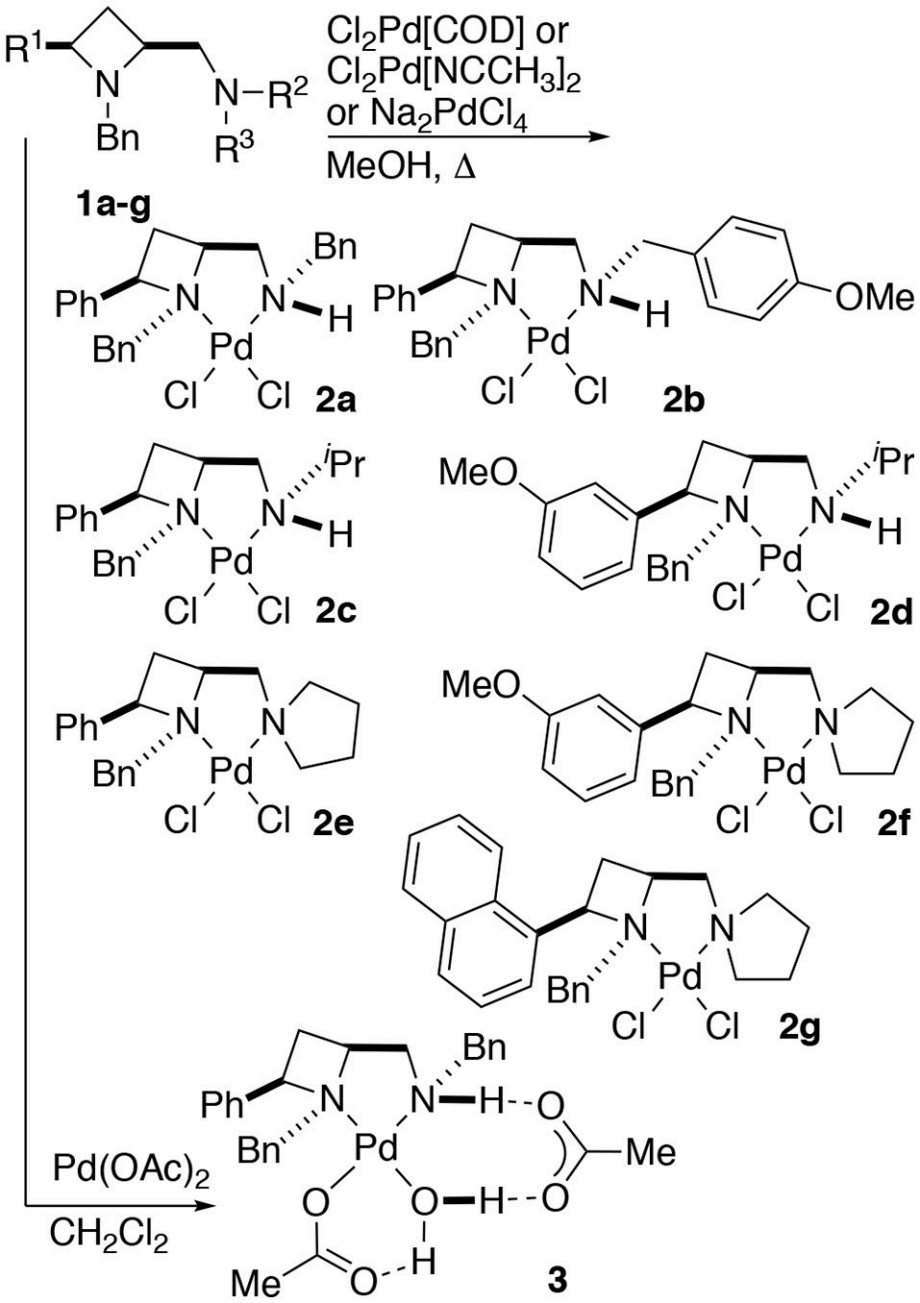
General protocol for the preparation of identified palladium(II) chloride **(2a–g)** and acetate **(3)** complexes of 2,4-*cis*-disubstituted azetidine *N,N*'-ligands.

**Figure 1:**
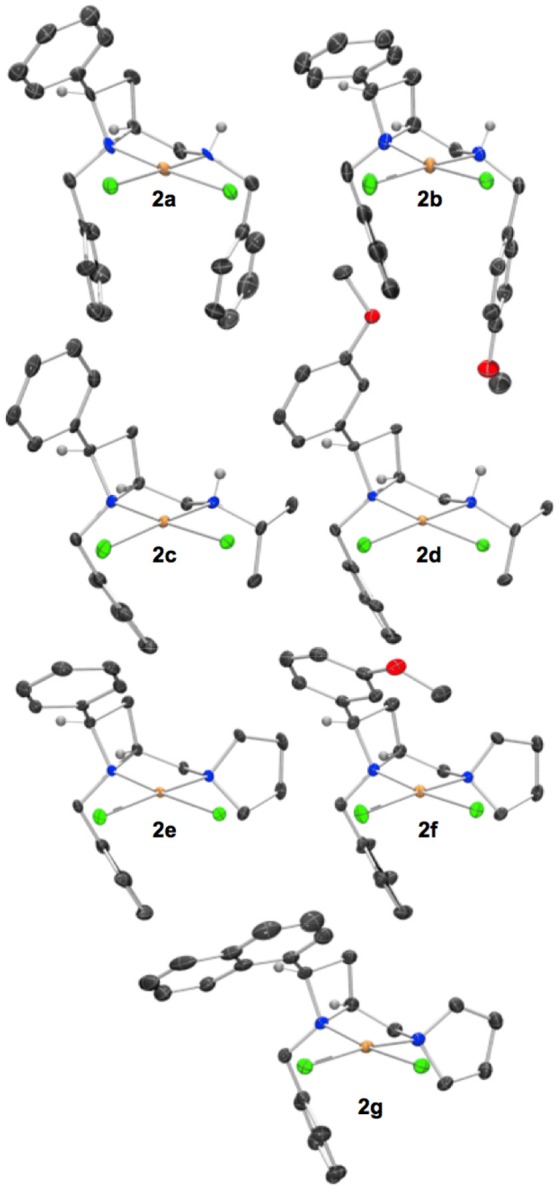
Representations of one molecule from the unit cells of the single crystal XRD structures of racemic complexes **2a**-**g**. Elipsoids plot at 50% probability, and rendered using Ortep-III for Windows and PovRay v3.7. For selected bond lengths, angles and torsions see ESI.

**Figure 2:**
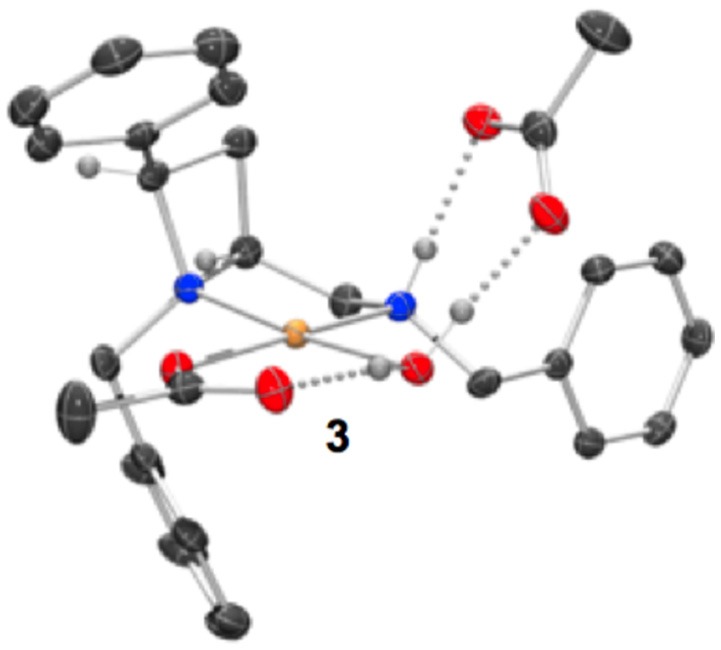
Representation of one molecule from the unit cell of the single crystal XRD structures of racemic complex **3**. Elipsoids plot at 50% probability, and rendered using Ortep-III for Windows and PovRay v3.7.

Treatment of a series of amino-substituted azetidines with various palladium(II) chloride derivatives afforded crystals suitable for analysis by single crystal XRD analysis in seven cases (Scheme 1). Racemic ligands **1a** to **g** were converted to the corresponding square planar palladium chloride complexes **2a** to **g**, by combination of various palladium(II) chloride sources and ligands in methanol and heating at reflux until ligand had been consumed by TLC analysis, see Figure 1 (and Supplementary Material) for representations of the palladium chloride complexes thus obtained. Treatment of ligand **1a** with palladium(II) acetate in dichloromethane afforded mono-nuclear palladium complex **3**, bearing a coordinated water molecule and displaying hydrogen bonding between the coordinated water molecule and the two acetate anions (coordinated to palladium and bridging ligand NH…H_2_O respectively), see Figure 2, for a representation of the crystal structure obtained for compound **3**.

Reactions were routinely conducted under anhydrous, oxygen-free conditions, but materials obtained from reactions were handled in air and no special precautions to exclude moisture or oxygen were taken. Crystals of palladium(II) chloride complexes (Figure 1) suitable for single crystal XRD analysis were typically obtained by crystallization from acetonitrile or acetonitrile/diethyl ether mixtures, pre-purification by flash chromatography or filtration through silica in dichloromethane/methanol mobile phase was sometimes advantageous. The XRD structures typically displayed single diastereoisomers of products as racemic mixtures. The figures of the main text show one molecule from the unit cell, displayed such that the stereochemistry of those drawn in the main text *match* permitting ready comparison of steric effects among the ligands compared.

Single crystals of complex **3** (Figure 2) were obtained by slow diffusion of hexane directly into the dichloromethane solvated reaction mixture.

**Scheme 2:**
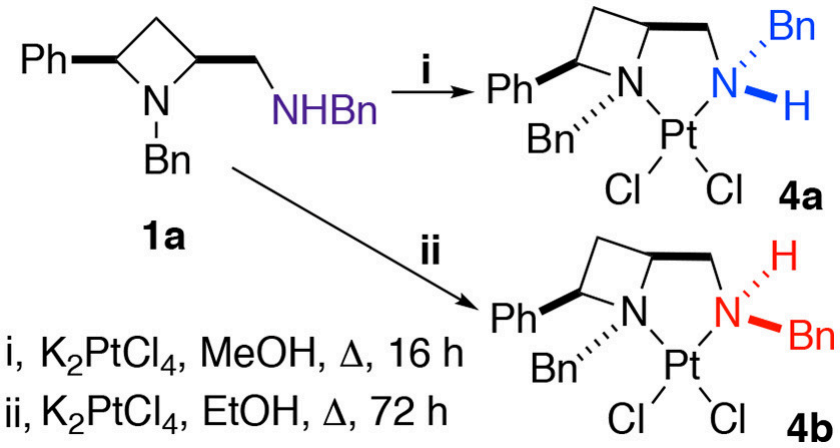
The protocol used to obtain crystals suitable for single crystal XRD analysis of *N*-diastereomeric racemic platinum(II) chloride complexes **4a** and **4b**.

The platinum(II) chloride complex **4a**, formed by heating ligand **1a** at reflux in methanol with potassium tetrachloroplatinate, was previously reported by us. We were intrigued when we later repeated the protocol, with ethanol as solvent, instead of methanol, and extended the reaction time somewhat (from 16 to 72 h), to find an alternative diastereoisomer, racemic **4b**, was formed, differing only by the coordinational chirality of the amine-part's nitrogen, both complexes are discussed in this report (Scheme 2). Gagné and co-workers previous discussed persistent nitrogen chirality in square planar diamine complexes of palladium(II) in their seminal work on the topic (Pelz and Gagné, 2003; Pelz et al., 2004), and their work should be consulted for discussion about complexes displaying chirality only as a result of nitrogen's stereogenicity as a result of coordination to a metal.

**Figure 3:**
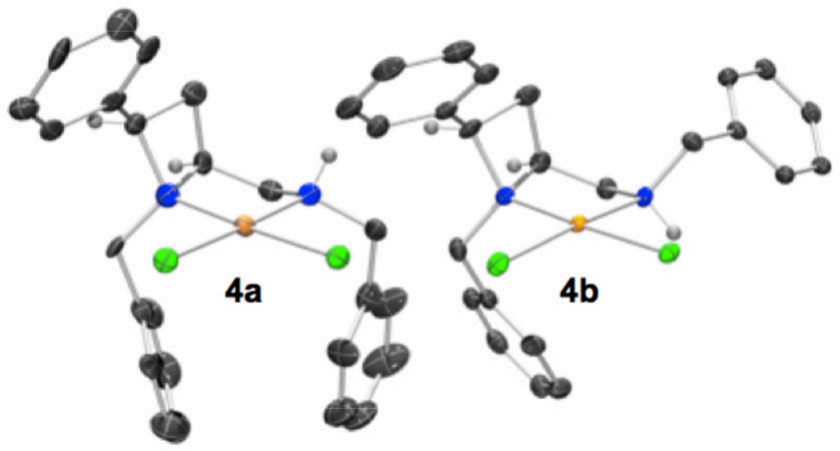
Representation of one molecule from each unit cell of racemic **4a** (left) and **4b** (right). The diastereoisomers drawn differ by inversion of the amine-centered nitrogen stereogenicity. The two diastereoisomers depicted are **4a** *N*_(*azet*)*R*_*N*_(*amin*)*R*_ (left) and **4b** *N*_(*azet*)*R*_*N*_(*amin*)*R*_ (right). Elipsoids plot at 50% probability, and rendered using Ortep-III for Windows and PovRay v3.7. For selected bond lengths, angles and torsions see ESI.

Crystallization from acetonitrile/diethyl ether or slow evaporation of an acetonitrile solution led to the isolation of crystals suitable for single crystal XRD analysis of **4a** and **4b** respectively, Figure 3.

**Figure 4:**
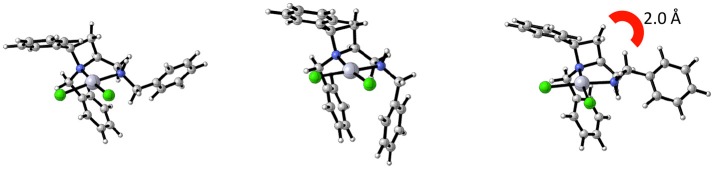
Calculated structural minima for **4a** (left) and **4b** (right), **4b** being 1.6 kcal/mol higher in relative free energy then **4a**. A crystal-structure-like accessible (0.8 kcal/mol higher in relative free energy) minima for **4a** depicted in the center.

Noting that platinum complex **4a** was formed under reflux in methanol (boiling point 64.7 °C) and the N-diastereomeric complex **4b** at reflux in ethanol (boiling point 78.4 °C) and over a longer time, it was reasoned that **4a** may be a kinetic product whilst **4b** the thermodynamic. If this were the case, there may be implications for the future use of such complexes in catalysis. Computational analysis revealed this assumption to be incorrect, importantly demonstrating that XRD analysis, especially in the case of this report where, in some cases, very few crystals were isolated from the bulk. Nevertheless, the computational analysis does demonstrate the constrained nature of the *cis*-azetidine architecture to be important in determining the underlying steric parameters around the coordination environment. The square planar N-diastereomeric complexes **4a** and **4b** were studied with density functional theory in order to establish the intrinsic thermodynamic preference. Calculations employed the M06-2X functional with the 6-31G^*^ basis set for all atoms apart from platinum atom which was treated with the LANL double zeta basis set and pseudopotential. Optimized geometries were subject to single point energy evaluations to evaluate the effects of solvation. Three conformations of the *N*-benzyl sidechain were studied, and energies of the lowest energy conformation reported. These calculations revealed that the lowest energy structure is that of **4a**. In these calculations, the preferred conformation of the sidechain is an extended one, Figure 4 (left). The alternative conformation [Figure 4 (center)], resembling that observed in the crystal structures is close in energy (0.8 kcal/mol) and so is readily accessible. The preferred conformation for **4b** is 1.6 kcal/mol higher in energy than the lowest energy conformation of **4a**, and its geometry as shown in Figure 4 (right) resembles the crystal structure of **4b** in Figure 3. The energy difference between **4a** and **4b** narrows slightly (by 0.01 kcal/mol) on changing from methanol solvation to ethanol. The origin of the preference for **4a** is likely the close approach required by the *N*-benzyl group to the hydrogen at the 2-position of the azetidine; this rigid system does not permit this close contact to be avoided. A table comparing and contrasting both crystallographically and computationally determined (*minima*) selected bond lengths, angles and torsions of 4a and 4b is included in the Supplementary Material. Good agreement between these data confirms the computed coordination environment matches that experimentally observed.

This computational analysis reveals that at equilibrium a mixture of **4a** and **4b** would consist mostly of **4a**. Yet in our slightly divergent preparation and perhaps more importantly slow crystallization conditions is was possible to favor crystalline deposition of the minor component (diffusion of diethyl ether into, vs. slow evaporation of acetonitrile).

**Figure 5:**
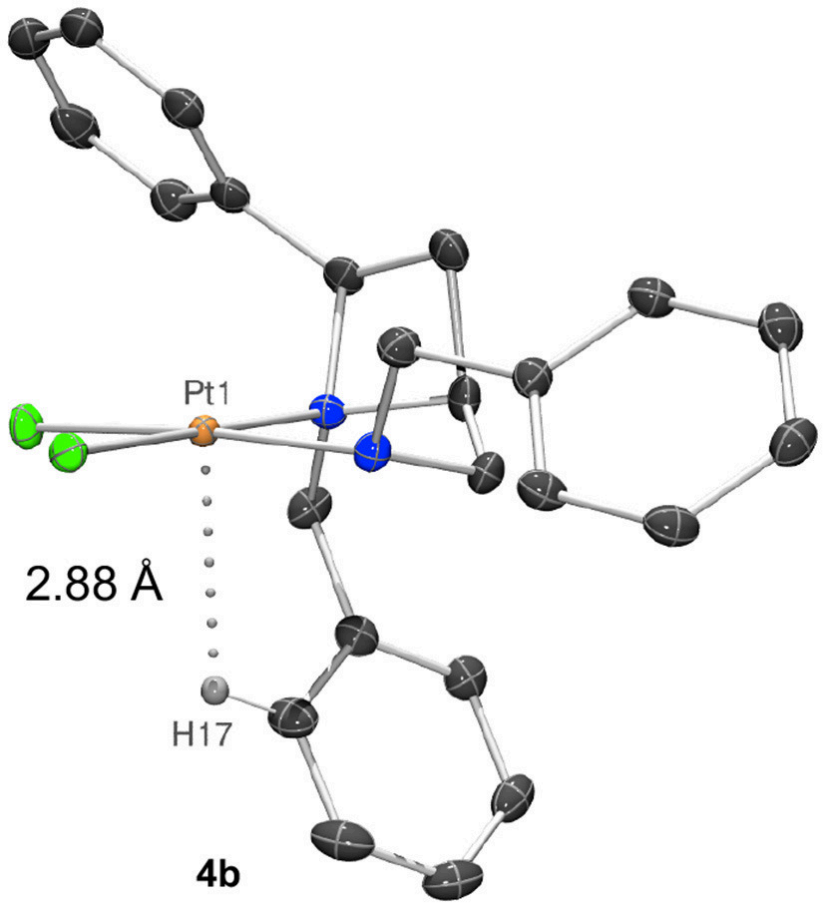
Representation of a molecule of **4b** showing a 2.8806 (3) Å distance between and aryl-H (H17) and the platinum(II) center (Pt1).

Closer inspection of the wider sphere about the metal atoms in the crystal structures shows that compound **4b** places the azetidine ring *N*-benzyl group closer to the platinum, potentially due to the space created by placing the exocyclic *N*-benzyl group on the opposite face of the square plane. This may be judged by a shortest aryl-H…Pt distance of 2.8806 (3) Å for **4b** (Figure 5) whereas aryl-H…Pt distances in **4a** are ≥3.00 Å (Hambley, 1998).

Whilst computationally reasoned to be primarily due to solid state, crystallization condition-dependent, phenomena we were interested to probe the consequences of the nitrogen atom stereogenicity upon the asymmetric, or more precisely the differing topological, properties of the cleft described by ligand **1a** in complexes **4a** and **4b**, as such the buried volume (%V_bur_) described by the ligand geometries was calculated. Inspired by the work of Nolan (Clavier and Nolan, 2010), Cavallo (Poater et al., 2009) and their co-workers the SambVca 2.0 tool was employed (Falivene et al., 2016), topological maps generated and their features compared^2^, to describe the steric constraints imposed about the exchangeable (halide) sites. PDB files containing one molecule of the single crystal X-ray diffraction-determined unit cell were uploaded to the SambVca 2.0 web-portal. The x axis being described as parallel with the N-N orientation of the square plane about the metal; the y axis lies perpendicular to the square plane of the metals' coordination; and the z axis bisects the both the two nitrogens and the two chlorides. The chlorides were removed in the web-portal and the %V_bur_ calculation run under otherwise default settings.

**Figure 6:**
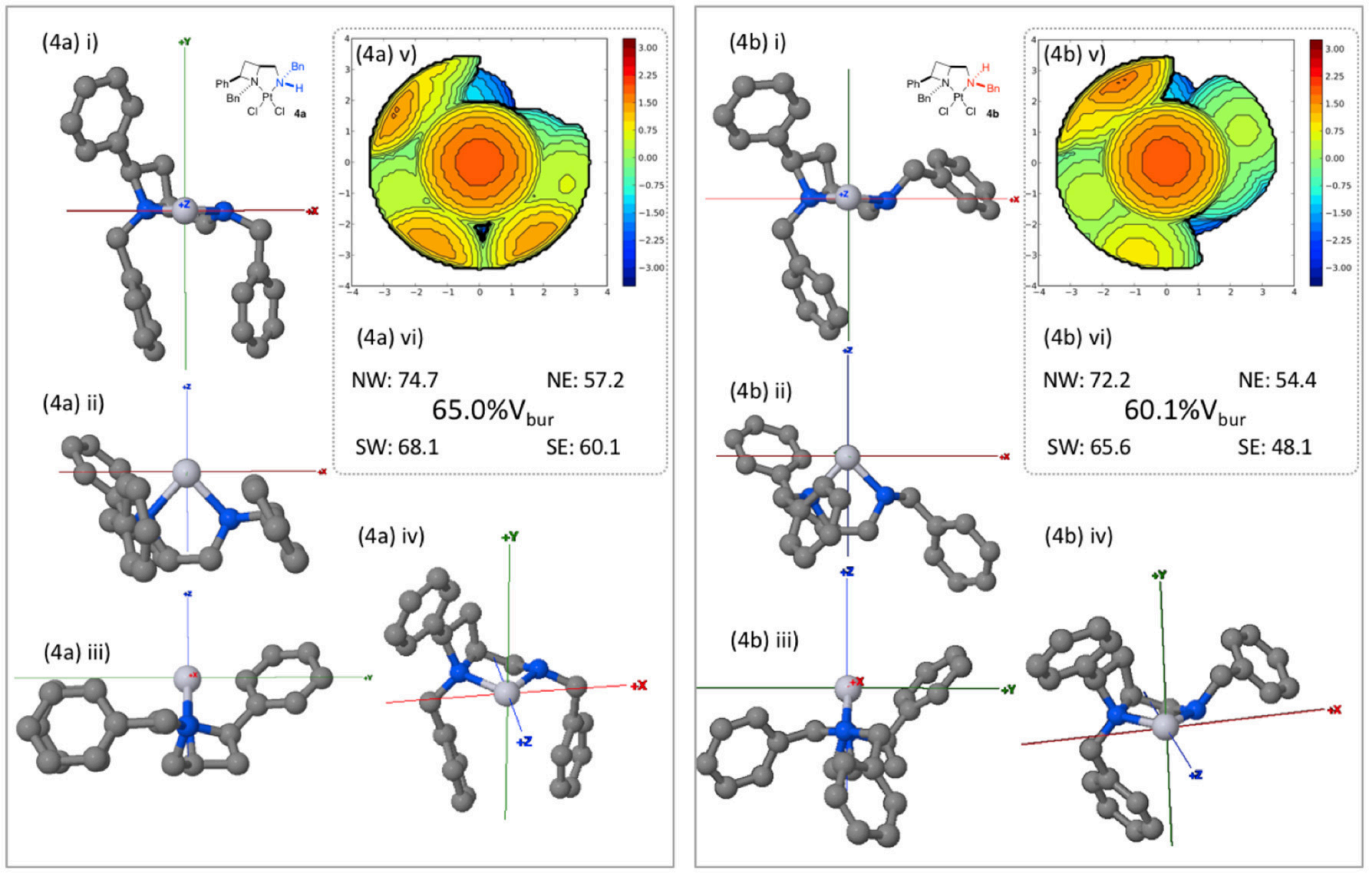
Burried volume analysis of ligand geometries in complexes **4a** and **4b**, conducted using online tool SambVca 2.0. (i-iv) Various orientations of the considered structures; (v) SambVca-generated %V_bur_ map ; and (vi) %V_bur_ and quadrantwise contributions.

Viewed from the *front* (down the z-axis as depicted in Figure 6) the diastereoisomeric complexes show similar steric constraints on the left upper and lower (NW and SW) quadrants. As expected the right side (Easterly) differs markedly between the diastereomeric complexes **4a** and **4b**, essentially leaving the NE and SE quadrants sterically unencumbered respectively. Thus, demonstrating the significance of nitrogen's coordinational chirality in defining the asymmetric environment a chiral ligand describes around a metal. In principle it may be reasoned that were the formed diastereomeric complexes stable and isolated as single enantiomers, that the same sense of carbon-centered stereogenicity could deliver opposite enantiomers of products in catalysis as a result of deviation in nitrogen-centered stereogenicity, a phenomenon warranting future further investigation. In order to permit direct comparison, steric maps of the percentage buried volume of all complexes of this report, generated by the SambVca 2.0 online tool, are included in the Supporting Information of this manuscript.

**Scheme 3:**
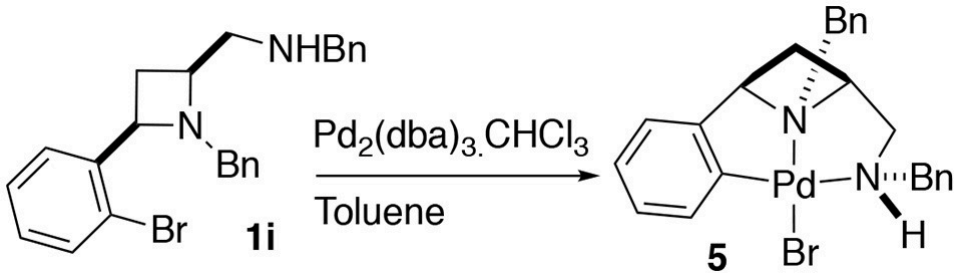
Oxidative addition of palladium(0) to **1i** to generate a palladium(II) *CNN*' metallocyclic complex.

**Figure 7:**
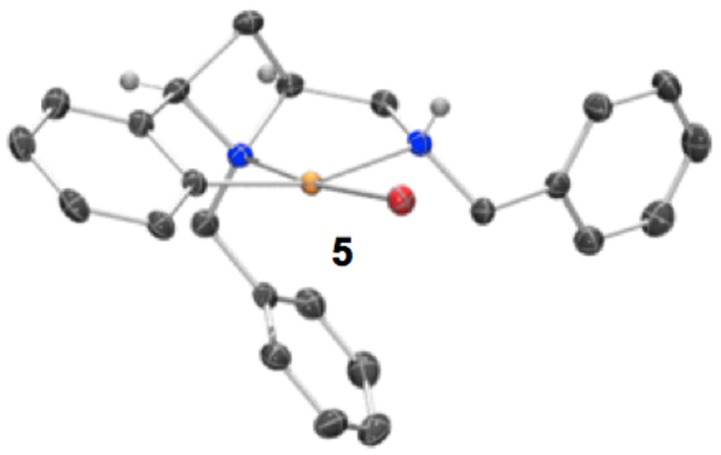
Representation of one molecule from the unit cell of the single crystal XRD structure of racemic complex **5**. Elipsoids plot at 50% probability, and rendered using Ortep-III for Windows and PovRay v3.7.

Having previously accessed *ortho* bromo ligand **1i** and having previously investigated oxidative addition of 10 metals to form NCN pincer complexes (Fossey and Richards, 2004; Fossey et al., 2007), it was reasoned that ligand **1i** may form a metallocyclic complex upon reaction with palladium(0), Scheme 3. To our delight a small amount of a *CNN*' metallocyclic complex (**5**) was identified, see Figure 7 for a representation of the crystal structure.

A unique opportunity provided by the 2,4-*cis*-disubstituted azetidine geometry is that a tridentate complexation of palladium is possible and concave ligand architecture is able to asymmetrically envelop the metal, and distort the metal coordination geometry away from an ideal square plane. The *trans* bond angles C-Pd-N and N-Pd-Br deviate from 180°, being 160.8° and 173.8° respectively, leading to slight pyramidalisation of the palladium(II) center (Figure 7).

**Scheme 4:**
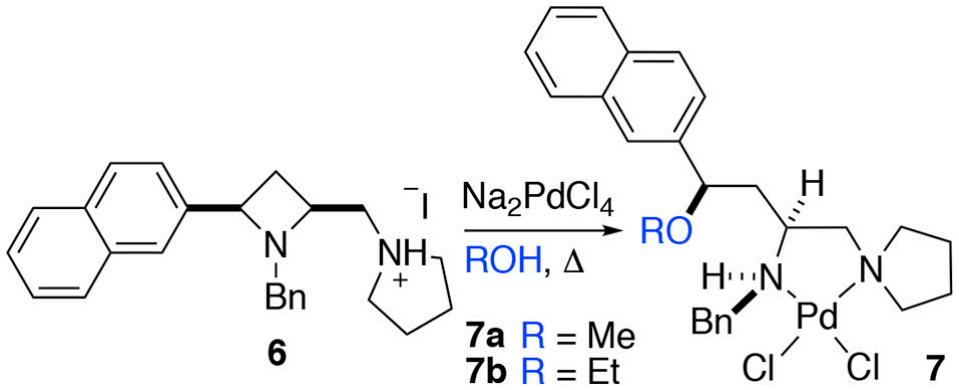
Methyl and ethyl ether complexes **7a** and **7b** resulting from azetidine ring opening of salt **6** and complexation to palladium(II) identified by single crystal XRD investigation.

**Figure 8:**
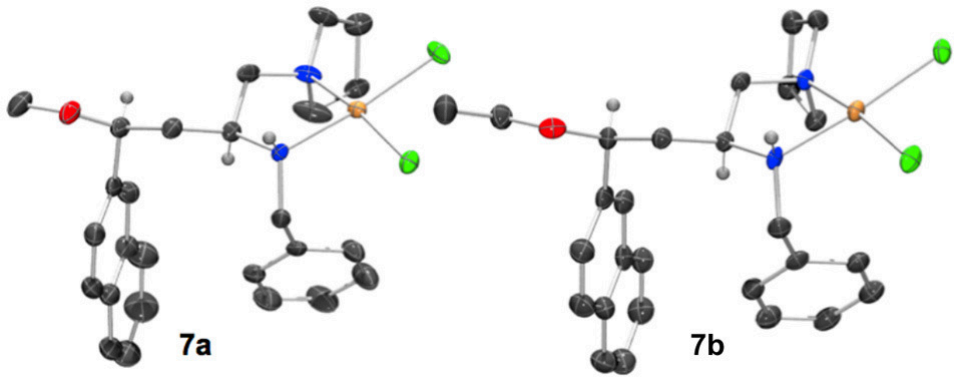
Representation of one molecule from the unit cells of the single crystal XRD structures of racemic complex **7a** and **7b**. Elipsoids plot at 50% probability, and rendered using Ortep-III for Windows and PovRay v3.7.

When a salt of an amino azetidine ligand (**6**) was exposed to sodium tetrachloropalladate and heated to reflux in methanol or ethanol ring-opened complexes (**7a** and **b**) were obtained, Scheme 4. Very few crystals were formed precluding analysis other than by single crystal XRD analysis alongside intractable material and it is not yet possible to say if all the ligand was consumed in this manner (Figure 8). But since prolonged heating of free-base ligands in methanol or ethanol with a metal source did not furnish any detectable ring-opened material it was reasoned that the small amount of crystals obtained resulted from acid promoted ring opening followed by coordination to palladium rather than the complex being unstable to the reaction conditions. This could be the subject of further future study, especially if the accessed ring opened complexes offer advantages in catalysis or other applications. The %V_bur_ of complexes **7a** and **b** are also calculated, and details are provided in the Supporting Information to allow for comparison amongst the complexes detailed in this manuscript.

The crystal structures of racemic complexes **7a** and **7b** display typical *NN'* binding modes in these palladium(II) chloride complexes, the stereogenic center on the carbon backbone imposes a *trans* stereochemical relationship upon the coordinationally chiral neighboring nitrogen atom (Fossey et al., 2005, 2008). The bulky groups point away from each other, and there is no evidence, in the solid state, of ancillary ether oxygen coordination. The crystals analyzed are single diastereoisomer racemates, meaning the ring opening step appears to have proceeded with high stereochemical fidelity, i.e., with clean inversion upon O-attack on the aryl substitute azetidinium ring stereogenic carbon (Gaertner, 1968; Leonard and Durand, 1968).

The synthesis of 2,4-*cis*-disubstituted azetidines by an iodocyclisation protocol used in the synthesis of azetidines **1a-g** can give rise to pyrrolidine by-products (see previous reports for details). In the case of the synthesis of complex **2d** a minor pyrrolidine impurity led to the formation and initial isolation of a complex derived from 2,4-*cis*-disubstituted pyrrolidine **8**.

**Scheme 5:**
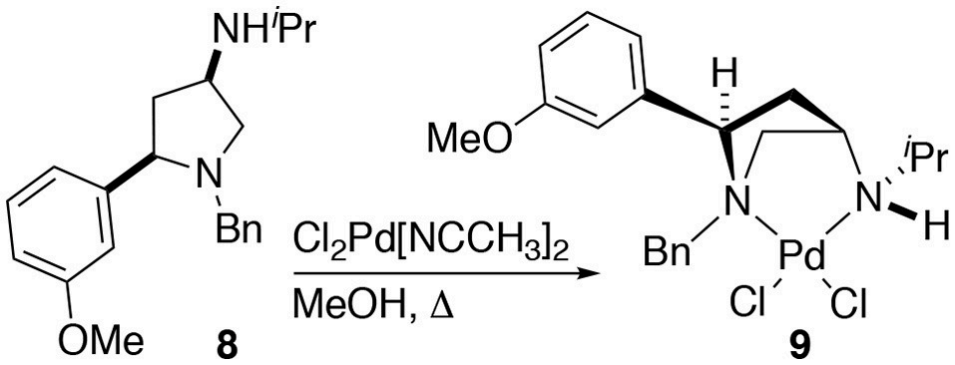
The formation of complex **9**. Namely through complexation of a minor impurity (**8**), leading to a crystalline material, one crystal, of a prominent crystal upon attempted crystallization, alter leading to **2d** after removal of the obtain crystal of **9**.

**Figure 9:**
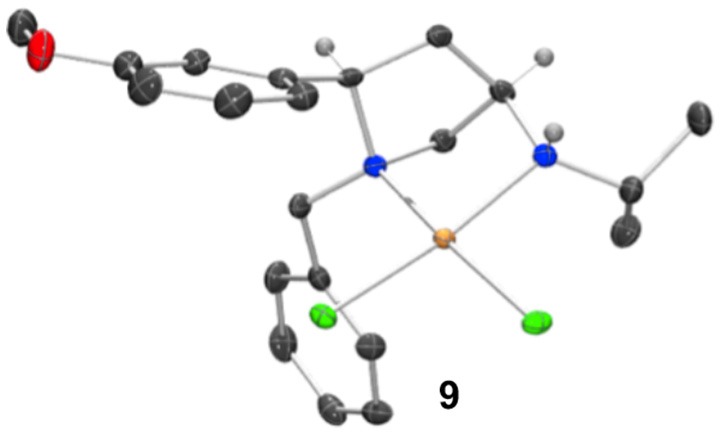
Representation of one molecule from the unit cell of the single crystal XRD structure of racemic complex **9**. Elipsoids plot at 50% probability, and rendered using Ortep-III for Windows and PovRay v3.7.

Whilst only a single crystal of the formed complex **9** was isolated and analyzed Scheme 5, as the minor component of the mixture, the complex itself displays some noteworthy features (Figure 9). Firstly, that even as a minor component, complex **9** readily formed a prominent crystal bodes well for the future targeted synthesis and isolation of it and its analogs. Secondly the *cis* relative stereochemistry across the 2,4-positions confers a geometry similar to, but subtly different from, azetidine analogs (compare **2d** with **9**). Importantly, in our previous work we showed how we can access both diastereoisomers of **8**-like pyrrolidines and a future planned programme of study will look in detail at the potential of 2,4-*cis*-pyrrolidines as scaffolds for catalyst construction (Feula et al., 2010, 2013).

**Figure 10:**
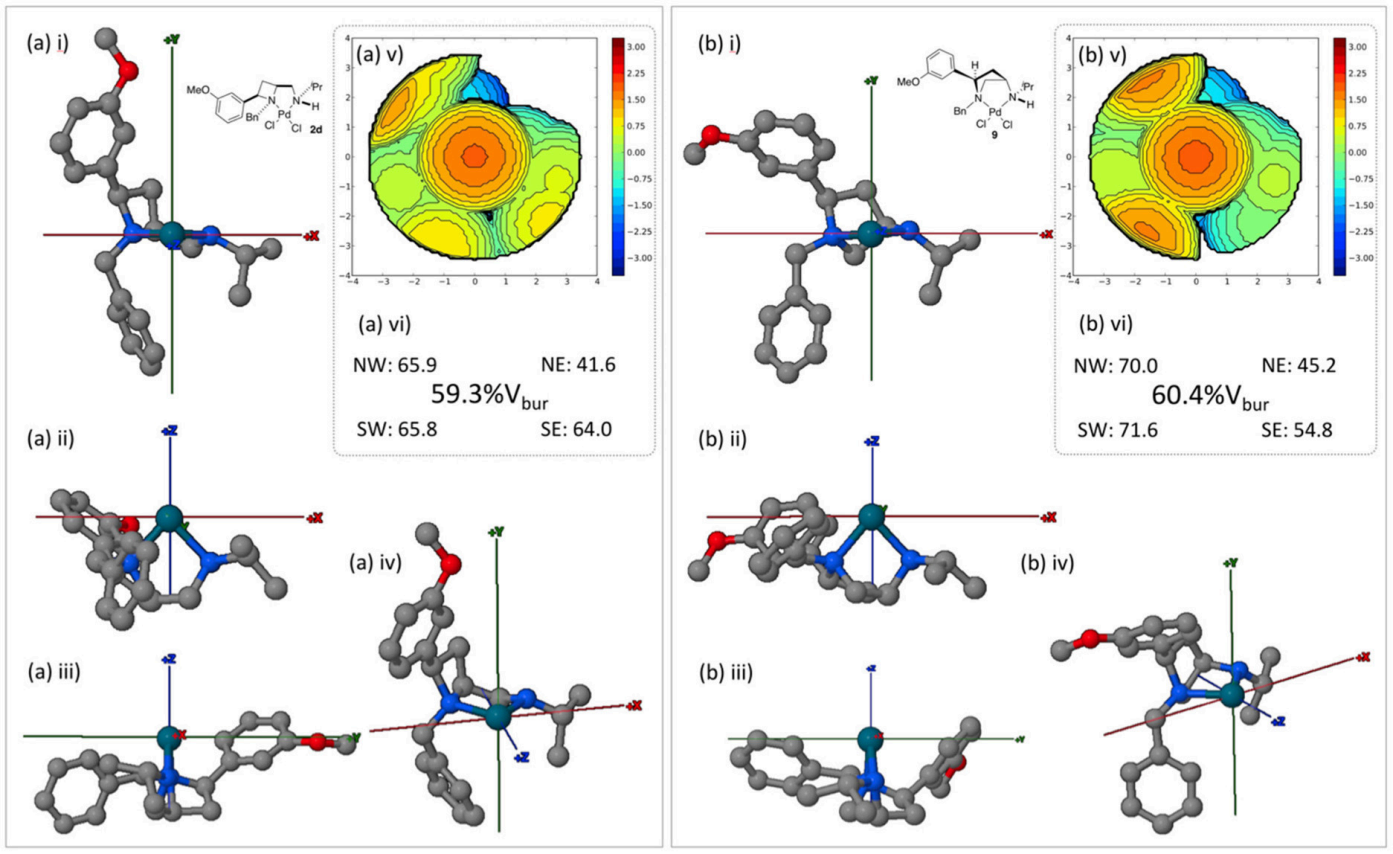
Burried volume analysis of ligand geometries in complexes **2d** and **9**, conducted using online tool SambVca 2.0. (i-iv) Various orientations of the considered structures; (v) SambVca-generated %V_bur_ map ; and (vi) %V_bur_ and quadrantwise contributions.

Since ligands **1d** and **8** are accessible form the same source, depending on divergent synthesis conditions (Feula et al., 2013), complexes **2d** and **9** were compared using the SambVca 2.0 online tool, Figure 10.

Under the same inspection protocols both the azetidine (**2d**) and pyrrolidine (**9**) complexes offer a similar level of steric constraint about their respective palladium(II) metal centers. However, the subtle impact of employing isomeric 4- and 5-membered heterocyclic ligands (**1d** and **8** respectively) can be witnessed in the buried volume differences between western and eastern hemispheres (as drawn). The average difference between the (western) left vs. (eastern) right increases from 13 to 21% across **2d** and **9** respectively. Thus, were single enantiomer asymmetric catalyst to be developed it may be expected that **9** offers greater steric discrimination across the described x axis. Whilst beyond the scope of this crystal structure report the displayed steric divergence between these isomeric ligands warrants further attention in an onward programme of research.

## Conclusions

Seven *N*,*N'*-palladium(II) chloride complexes, one *N*,*N'*-palladium(II) acetate complex of 2,4-*cis*-azetidines where prepared and analyzed by single crystal XRD. The racemic ligands adopted a single diastereoismer form upon coordination to palladium the same chirality at nitrogen. In the palladium(II) acetate complex a coordinated water molecule and H-bonding acetates formed the identified complex. Two platinum(II) chloride *N*,*N'*-complexes of 2,4-*cis*-azetidines where prepared and analyzed by single crystal XRD, and two diastereoisomers were generated upon amine coordination to platinum (under different preparation conditions). Computational analysis revealed which diastereoisomer was more stable and provided a rationale for why this is the case, and the %V_bur_ described by the diastereomeric coordination geometry was examined. A *CNN*' metallocyclic complex was prepared by oxidative addition of palladium(0) to an *ortho* bromo 2,4-*cis-*disubstituted azetidine and its crystal structure displays a slightly pyramidalized metal-ligand orientation. Ligand salts were not suitable for the synthesis of azetidine complexes, instead leading to *N*,*N'* complexation of stereospecifically ring-opened congeners. A minor, pyrrolidine, impurity in an azetidine ligand sample led, initially, to the formation of a highly crystalline complex, identified by single crystal XRD, as well as (later) from the same sample the expected azetidine complex. The isomeric azetidine and pyrrolidine complexes, characterized by single crystal XRD, were studied using the SambVca 2.0 online tool and their steric parameters contrasted revealing the potential for both azetidine and pyrrolidine ligands in future catalytic applications^3^.

## Author contributions

AF developed the ligand synthesis protocol (reported elsewhere) and synthesized some *cis*-aminoazetidines and prepared XRD-quality crystals of the first *N*,*N*'-platinum and -palladium complexes of them, the first organometallic *CNN*'-palladium azetidine complex was also prepared, AF co-wrote sections of the manuscript; JF is the lead and corresponding author who conceived and led the project, JF conceived the project and co-wrote the manuscript; AL: calculated the free energies of the diastereomeric platinum complexes and wrote the aspects of the manuscript pertaining to that aspect; LM recorded, analyzed and interpreted some of the X-ray crystallography data of this report and has oversight of all of the X-ray crystallography data herein. LM conducted training of AY, in order for AY to carry out XRD analysis, LM co-wrote aspects of the report pertaining to XRD data collection and analysis; AY synthesized some *cis*-aminoazetidines and prepared XRD-quality crystals of *N*,*N*'-platinum and -palladium complexes of them. AY obtained the crystal of the *cis*-pyrrolidine palladium complex and probed the solvent-mediated ring opening of an azetidine salt during a palladium complexation protocol. AY prepared ligands and complexes, analyzed and interpreted some of the XRD data in this report and co-wrote aspects of the report. All authors made critical contributions to the report, gave significant input into the report writing process and analysis of data herein.

### Conflict of interest statement

The authors declare that the research was conducted in the absence of any commercial or financial relationships that could be construed as a potential conflict of interest.

## Footnotes

^1^The strucutre of 4a was published previously as compound see (*rac*)-7 in reference 4a. With a UK National Crystallography Service Code: **2010src0915**.

^2^The crystal structure and SambVca analysis of complex **4a** was already reported by us in a paper pertaining to copper-cataylzed Henry reactions. Data for compound **4a** is included here to permit facile comparison amongst the platinum and palladium complexes of this report.

^3^A preprint version of this manuscript was submitted to ChemRxiv prior to the peer revewing process.
