# Supplementary material for: Palladium and Platinum 2,4-cis-amino Azetidine and Related Complexes
Source: Front Chem. 2018 Jun 21;6:211. doi: 10.3389/fchem.2018.00211 (PMC6021532; doi:10.3389/fchem.2018.00211)
Supplement: Supplementary file 1 [file Data_Sheet_1.PDF]

Supplementary Information:

Palladium and platinum 2,4-cis-amino azetidine and related complexes

Akina Yoshizawa,[(a)] Antonio Feula,[(a)] Andrew G. Leach,[(b)] Louise Male[(c)] and John S. Fossey[(a)*]

(a) School of Chemistry, University of Birmingham, Edgbaston, Birmingham, West Midlands, B15 2TT, UK.

(b) School of Pharmacy and Biomolecular Sciences, Liverpool John Moores University, Byrom Street, Liverpool L3 3AF, UK.

(c) X-Ray Crystallography Facility, School of Chemistry, University of Birmingham, Edgbaston, Birmingham, West Midlands, B15 2TT, UK.

Table of contents

Table of contents 1
General methods 2
Percent buried volume 2
Experimental 4
Computational tables of coordinates 7
4a lowest energy N-benzyl rotamer 7
4a second lowest energy N-benzyl rotamer 9
4a highest energy N-benzyl rotamer 10
4b lowest energy N-benzyl rotamer 11
4b second lowest energy N-benzyl rotamer 12
4b highest energy N-benzyl rotamer 13
Computational and crystallographically determined bond lengths, angles and torsion comparison 15
Single crystal X-ray diffraction information 15
Crystal structure determination of 2a 16
Crystal structure determination of 2b 16
Crystal structure determination of 2c 17
Crystal structure determination of 2d 17
Crystal structure determination of 2e 17
Crystal structure determination of 2f 18
Crystal structure determination of 2g 18
Crystal structure determination of 3 18
Crystal structure determination of 4a. 18
Crystal structure determination of 4b 19
Crystal structure determination of 5 19
Crystal structure determination of 7a 20
Crystal structure determination of 7b 20
Crystal structure determination of 9 20
Further information added to clarify issues surrounding symmetry 20
Tables of selected bond lengths, angle and torsions from XRD analysis 21
References 23

General methods

Reagents were used as purchased from commercial suppliers without further purification. Anhydrous solvents were dispensed from a solvent purification system (SPS), monitored by Karl-Fisher titrations for water levels. Proton NMR spectra were recorded at 300 MHz on a Bruker AVIII300 NMR spectrometer or at 400 MHz on a Bruker AVIII400 NMR spectrometer. All ^{13}C NMR spectra are proton decoupled and recorded at 101 MHz on a Bruker AVIII400 NMR spectrometer or at 75.5 MHz on a Bruker AVIII300 NMR spectrometer. Proton NMR chemical shifts are reported in ppm relative to TMS (δ 0.00) or residual solvent signals, ^{13}C NMR shifts relative to chloroform (δ 77.36). Coupling constants *J* are given in Hertz (Hz). Melting points were carried out in triplicate and an average of the values taken and reported as a range using Stuart™ digital melting point apparatus (SMP10). Infrared spectra were recorded on a PerkinElmer 100 FT-IR spectrometer at room temperature. The majority of column chromatography was carried out using a Combiflash R*f* 200i (silica 4-12 g column). Elemental analysis were recorded on a CE Instruments EA1110 elemental analyser, measurements of each sample were recorded twice and the average used. All calculations were performed in Gaussian09. Minima and transition states were confirmed by computation of second-derivatives.

Percent buried volume

Graphical summaries of the percentage buried volume determinations using the SambVca 2.0 online tool.[1] Determined from experimentally derived PDB files determined by single crystal X-ray diffraction analysis.

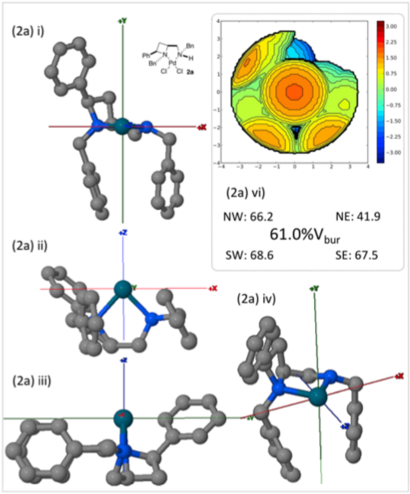

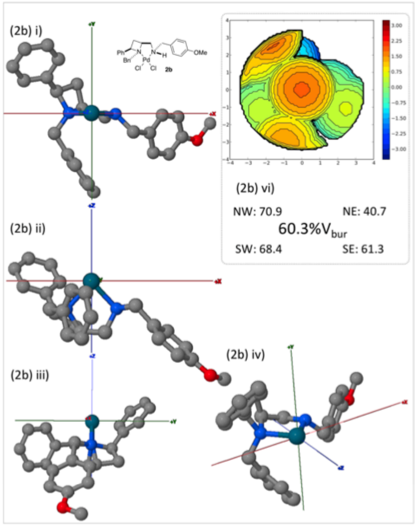

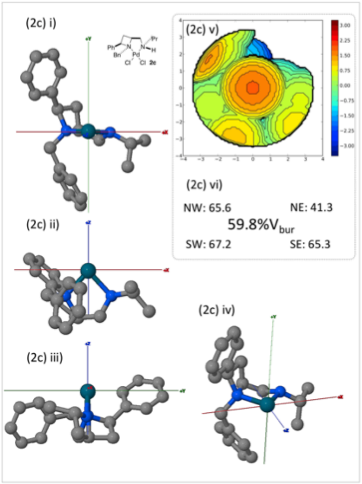

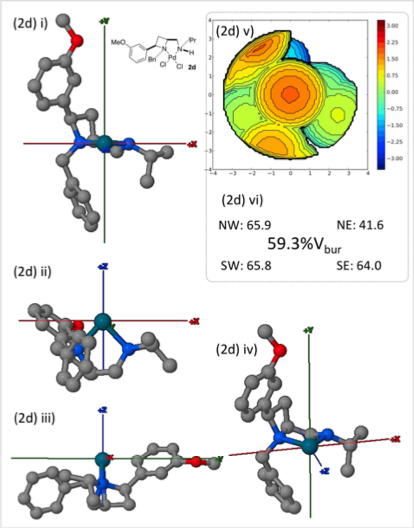
(2d) i)
(2d) v)
(2d) vi)
NW: 65.9
NE: 41.6
59.3%V$_{bur}$
SW: 65.8
SE: 64.0
(2d) ii)
(2d) iv)
(2d) iii)

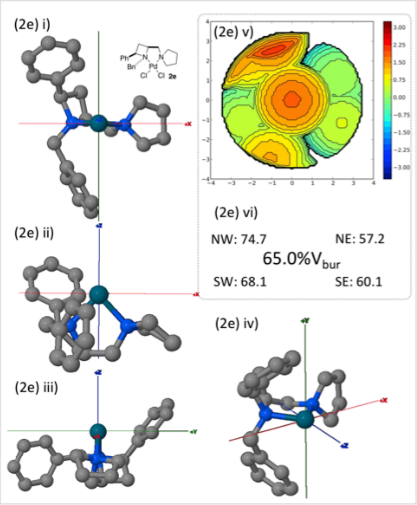
(2e) i)
(2e) v)
(2e) vi)
NW: 74.7
NE: 57.2
65.0%V$_{bur}$
SW: 68.1
SE: 60.1
(2e) ii)
(2e) iv)
(2e) iii)

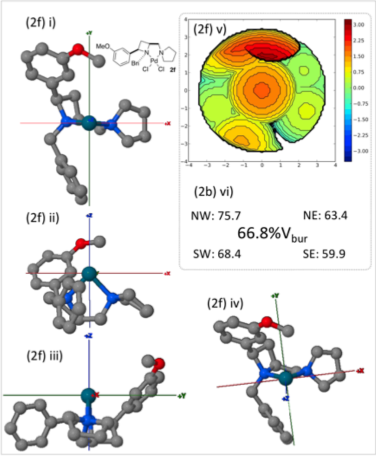
(2f) i)
(2f) v)
(2b) vi)
NW: 75.7
NE: 63.4
66.8%V$_{bur}$
SW: 68.4
SE: 59.9
(2f) ii)
(2f) iv)
(2f) iii)

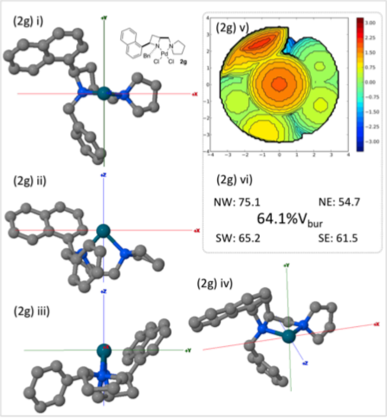
(2g) i)
(2g) v)
(2g) vi)
NW: 75.1
NE: 54.7
64.1%V$_{bur}$
SW: 65.2
SE: 61.5
(2g) ii)
(2g) iv)
(2g) iii)

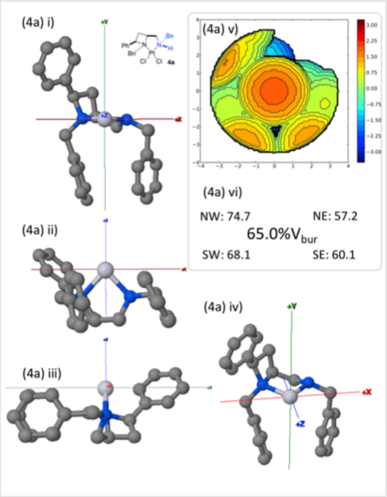
(4a) i)
(4a) v)
(4a) vi)
NW: 74.7
NE: 57.2
65.0%V$_{bur}$
SW: 68.1
SE: 60.1
(4a) ii)
(4a) iv)
(4a) iii)

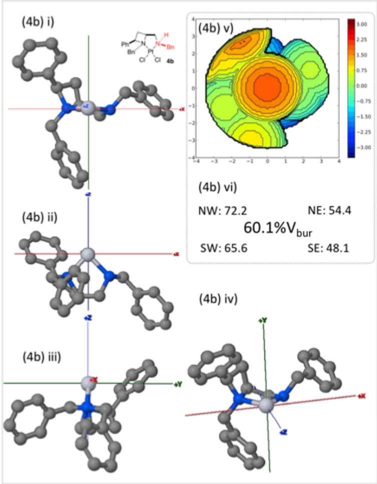
(4b) i)
(4b) v)
(4b) vi)
NW: 72.2
NE: 54.4
60.1%V$_{bur}$
SW: 65.6
SE: 48.1
(4b) ii)
(4b) iv)
(4b) iii)

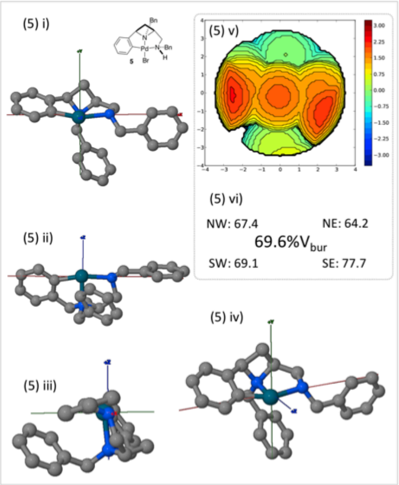

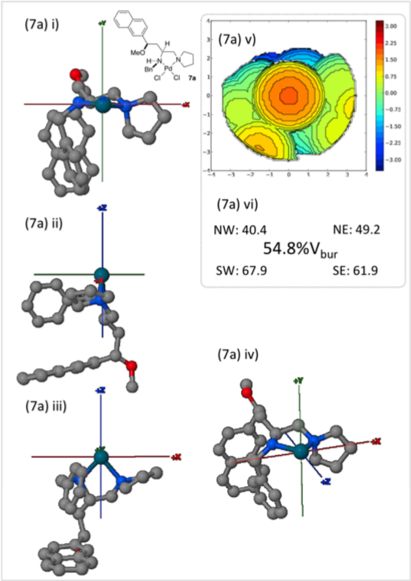

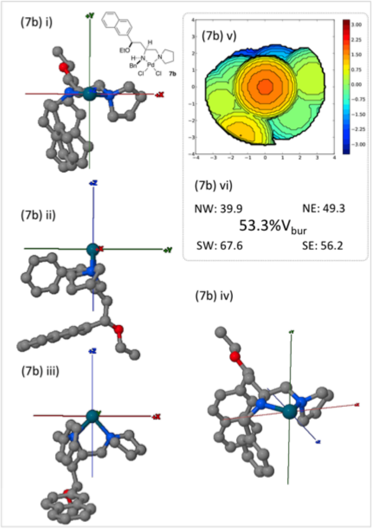

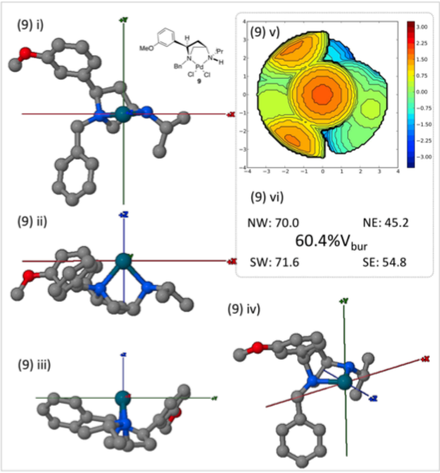

Experimental

Compound 2a: Under a nitrogen atmosphere, sodium tetrachloropalladate (85.9 mg, 0.292 mmol) was added to a solution of ethanol (20 mL) and compound **1a** (100 mg, 0.292 mmol). The mixture was stirred for 16 hours at reflux, after which the solvent was removed *in vacuo* and purified by flash column chromatography (R_f = 0.36, silica CH_2Cl_2:MeOH 95:5). Yellow/orange solid (103 mg, 0.198 mmol) 68% yield. Orange block crystals suitable for single crystal X-ray diffraction analysis were obtained by slow evaporation of concentrated acetonitrile solution. Mpt: 222-223 °C; IR (solid) ν_{max} = 3151 (m), 3065 (w), 3029 (w), 1497 (m), 1451 (m), 1360 (w), 1204 (w), 1075 (w), 1024 (m), 900 (w), 880 (w), 810 (w), 759 (s), 742 (s), 702 (s), 691 (s) cm^{-1}; MS TOF ES+ (*m/z*): 449.2 $[M-2Cl]^{2+}$, 1004.3 $[2M-Cl]^{+}$; Elemental analysis

calcd (%) for $C_{24}H_{26}Cl_2N_2Pd$ (H_2O + 0.5 CH_3CN): C 53.78, H 5.33, N 6.27. Found: C 53.75, H 5.38, N 6.30.

Compound 2b: Under a nitrogen atmosphere, sodium tetrachloropalladate (43 mg, 0.15 mmol) was added to a solution of methanol and compound **1b** (60 mg, 0.16 mmol). The mixture was stirred for 16 hours at room temperature, after which the solvent was removed *in vacuo* and the product purified by crystallisation (CH_3CN:Et_2O). Yellow crystals (50 mg) 57% yield. ^1H NMR (δ; 300 MHz, $CDCl_3$); 1.21-1.31 (1H, m), 1.75 (1H, dd, *J* 13.9 & 5.3), 2.02-2.10 (1H, m), 3.11 (1H, dd, *J* 13.6 & 9.6), 3.23 (1H, d, *J* 11.0), 3.37 (1H, d, *J* 11.7), 3.45-3.53 (1H, m), 3.70 (3H, s), 4.19 (1H, dd, *J* 10.9 & 7.2), 5.00 (2H, ABq, J_{AB} 11.7), 5.29 (1H, br), 6.67 (2H, d, *J* 8.6), 6.91-6.93 (3H, m), 7.05 (2H, d, *J* 8.6), 7.50-7.53 (3H, m), 7.74-7.77 (2H, m), 8.28-8.31 (2H, m); ^{13}C NMR (δ; 100 MHz, $CDCl_3$), 24.42 (CH_2), 55.09 (CH_2), 55.28 (CH_3), 57.80 (CH_2), 66.38 (CH_2), 67.26 (CH), 75.83 (CH), 114.19 (CH), 115.40 (C), 126.23 (C), 128.37 (CH), 128.78 (CH), 129.12 (CH), 129.33 (CH), 130.28 (CH), 131.61 (CH), 132.04 (CH), 133.48 (C), 134.65 (C). (*m/z*): (ES^+) 515.4, 477.4.

Compound 2c: Under a nitrogen atmosphere, sodium tetrachloropalladate (50.0 mg, 0.170 mmol) was added to a solution of methanol (20 mL) and compound **1c** (150 mg, 0.171 mmol). The mixture was stirred for 16 hours at reflux, after which the solvent was removed in vacuo and orange block crystals suitable for single crystal X-ray analysis ware obtained by cooling an acetonitrile and diethyl ether solution to 4 °C, (25.0 mg, 0.053 mmol) 31% yield. Mpt: 198-199 °C; MS TOF ES+ (m/z): 435.1 $[M-Cl]^+$.

Compound 2d: Under a nitrogen atmosphere, sodium tetrachloropalladate (45.3 mg, 0.154 mmol) was added to a solution of ethanol (20 mL) and compound **1d** (50.0 mg, 0.154 mmol). The mixture was stirred for 16 hours at reflux, after which the solvent was removed *in vacuo* and orange block crystals suitable for single crystal X-ray analysis ware obtained by cooling an acetonitrile and diethyl ether solution to 4 °C, (55.0 mg, 0.110 mmol) 71% yield. From which a single prominent crystal of compound **9** was collected (see compound **9**) and the residual material was determined to be compound **2d**. Mpt:196-198 °C (decomposition); IR (solid) ν_{max} = 3147 (s), 3065 (w), 3014 (w), 2981 (w), 2966 (w), 2930 (w), 2876 (w), 2837 (w), 1607 (m), 1583 (s), 1489 (s), 1452 (s), 1431 (s), 1395 (m), 1336 (m), 1284 (s), 1244 (s), 1223 (m), 1164 (s), 1143 (s), 1052 (m), 1039 (s), 986 (m), 948 (s), 892 (m), 880 (s), 791 (s), 754 (s), 707 (s), 694 (s) cm^{-1}; MS TOF ES+ (*m/z*): 523.1 $[M+Na]^+$; Elemental analysis calcd (%) for $C_{21}H_{28}Cl_2N_2OPd$: C 50.27, H 5.62, N 5.58. Found: C 50.31, H 5.65, N 5.68.

Compound 2e: Under a nitrogen atmosphere, sodium tetrachloropalladate (96.0 mg, 0.326 mmol) was added to a solution of ethanol (20 mL) and compound **1e** (100 mg, 0.326 mmol). The mixture was stirred

for 6 hours at reflux, after which the solvent was removed *in vacuo* and orange block crystals suitable for single crystal X-ray analysis ware obtained by slow evaporation of a concentrated acetonitrile solution, (44.0 mg, 0.091 mmol) 28% yield. Mpt: 215-216 °C; IR (solid) ν_{max} = 2970 (m), 2882 (w), 1513 (w), 1496 (m), 1456 (m), 1442 (m), 1360 (w), 1329 (w), 1245 (w), 1209 (m), 1068 (w), 1030 (m), 935 (m), 900 (m), 818 (m), 791 (s), 769 (s), 776 (s), 746 (s), 700 (s) cm^{-1}; MS TOF ES+ (*m/z*): 447.2 [M-Cl]$^+$, 894.4 [2M-2Cl]$^+$, 929.3 [2M-Cl]$^+$.

Compound 2f: Under a nitrogen atmosphere, sodium tetrachloropalladate (43.7 mg, 0.149 mmol) was added to a solution of ethanol (10 mL) and compound **1f** (50.0 mg, 0.149 mmol). The mixture was stirred for 16 hours at reflux, after which the solvent was removed *in vacuo* and orange block crystals suitable for single crystal X-ray analysis ware obtained by cooling an acetonitrile and diethyl ether solution to 4 °C, (66.0 mg, 0.128 mmol) 86% yield. Mpt: 199-200 °C; IR (solid) ν_{max} = 3005 (w), 2967 (w), 2927 (w), 2885 (w), 2837 (w), 1602 (s), 1492 (s), 1468 (s), 1453 (s), 1433 (s), 1391 (m), 1349 (m), 1319 (m), 1300 (w), 1279 (s), 1260 (s), 1222 (w), 1168 (s), 1089 (m), 1055 (m), 1033 (s), 1018 (s), 939 (s), 907 (m), 883 (m), 849 (s), 800 (s), 788 (s), 745 (s), 701 (s) cm^{-1}; MS TOF ES+ (*m/z*): 535.1 [M+Na]$^+$; Elemental analysis calcd (%) for $C_{22}H_{28}Cl_2N_2OPd$: C 51.43, H 5.49, N 5.45. Found: C 51.37, H 5.49, N 5.66.

Compound 2g: Under a nitrogen atmosphere, sodium tetrachloropalladate (82.5 mg, 0.280 mmol) was added to a solution of ethanol (20 mL) and compound **1e** (100 mg, 0.280 mmol). The mixture was stirred for 16 hours at reflux, after which the solvent was removed in vacuo and orange block crystals suitable for single crystal X-ray analysis ware obtained by slow evaporation of a concentrated acetonitrile solution, (121 mg, 0.227 mmol) 81% yield. Mpt: 194-196 °C (decomposition); IR (solid) ν_{max} = 3053 (w), 2968 (w), 2927 (w), 1600 (w), 1513 (w), 1495 (w), 1455 (m), 1396 (w), 1363 (w), 1328 (m), 1246 (w), 1211 (w), 1158 (w), 937 (m), 899 (m), 861 (m), 791 (s), 786 (s), 746 (s), 704 (s) cm^{-1}; MS TOF ES+ (*m/z*): 497.1 [M-Cl]$^+$; Elemental analysis calcd (%) for $C_{25}H_{28}Cl_2N_2Pd$: C 56.25, H 5.29, N 5.25. Found: C 56.19, H 5.43, N 5.35.

Compound 3: A solution of **1a** (100 mg, 0.292 mmol) in dichloromethane (1 mL) was added to a suspension of palladium(II)acetate (65.6 mg, 0.292 mmol) in dichloromethane (1 mL) and stirred for five hours. Pale yellow crystals suitable for single crystal X-ray analysis ware obtained by slow diffusion of *n*-hexane into this mixture over two weeks. Mpt: 114-115 °C; IR (solid) ν_{max} = 3045 (w), 1575 (m), 1498 (m), 1453 (s), 1398 (s), 1335 (m), 1284 (m), 1160 (m), 1029 (s), 914 (m), 810 (m), 768 (m), 750 (s), 732 (m), 702 (s), 675 (s) cm^{-1}; MS TOF ES+ (*m/z*): 589.4 [M+Na]$^+$; Elemental analysis calcd for $C_{28}H_{32}O_4N_2Pd \cdot CH_2Cl_2$: C 53.43, H 5.26, N 4.30. Found: C 53.85, H 5.57, N 4.52.

Compound 4a: Under a nitrogen atmosphere, K_2PtCl_4 (110 mg, 0.26 mmol) was added to a solution of methanol and ligand **1a** (100 mg, 0.29 mmol). The mixture stirred for 16 hours at 60 °C after which the solvent was removed *in vacuo* and the product purified by crystallisation (CH_3CN:Et_2O). Yellow crystals, (115 mg) 65% yield. (*m/z*): (ES^+) 572.4, 536.1.

Compound 4b: Under a nitrogen atmosphere, sodium tetrachloroplatinate (101 mg, 0.243 mmol) was added to a solution of ethanol (20 mL) and compound **1a** (83.2 mg, 0.243 mmol). The mixture was stirred for 72 h at reflux, after which the solvent was removed *in vacuo*, crystals suitable for single crystal X-ray analysis ware obtained by slow evaporation of a concentrated acetonitrile solution. Pale yellow solid (66.0 mg, 0.108 mmol) 44% yield. Mpt: 215-216 °C; IR (solid) ν_{max} = 3146 (m), 3026 (w), 1497 (m), 1455 (m), 1363 (w), 1255 (w), 1170 (w), 1075 (w), 1021 (m), 881 (w), 811 (w), 759 (s), 744 (s), 701 (s), 690 (s) cm^{-1}; MS TOF ES+ (*m/z*): 572 $[M-Cl]^+$; Elemental analysis calcd (%) for $C_{24}H_{26}Cl_2N_2Pt$:C 47.38, H 4.31, N 4.60. Found: C 47.36, H 4.29, N 4.43.

Compound 5: Under a nitrogen atmosphere, $Pd_2(dba)_2$ (136 mg, 0.24 mmol) was added to a toluene solution of amino-azetidine **1i** (50 mg, 0.12 mmol), the mixture stirred for 16 h at 100 °C. The solvent was removed *in vacuo* and the product purified by crystallisation (EtOAc:Et_2O). Yellow crystals (46 mg) 73% yield. High-resolution MS (*m/z*) calcd. for formula $C_{24}H_{25}N_2Pd^+$ $[M-Br]^+$: 447.1053; found: 447.1060.

Compound 7a: Under a nitrogen atmosphere, sodium tetrachloropalladate (30.3 mg, 0.103 mmol) was added to a solution of methanol (10 mL) and **6** (50.0 mg, 0.03 mmol). The mixture was stirred for 16 hours at reflux, after which the solvent was removed *in vacuo* and orange needle crystals suitable for single crystal X-ray analysis ware obtained by slow evaporation of a concentrated acetonitrile solution.

Compound 7b: Under a nitrogen atmosphere, sodium tetrachloropalladate (30.3 mg, 0.103 mmol) was added to a solution of ethanol (10 mL) and **6** (50.0 mg, 0.103 mmol). The mixture was stirred for 16 hours at reflux, after which the solvent was removed *in vacuo* and orange needle crystals suitable for single crystal X-ray analysis ware obtained by cooling an acetonitrile and diethyl ether solution to 4 °C.

Compound 9: See compound **2d**.

Computational tables of coordinates

4a lowest energy N-benzyl rotamer

SCF energy= -2078.68444732
Zero-point correction= 0.458793
Thermal correction to Energy= 0.485343

Thermal correction to Enthalpy= 0.486287
Thermal correction to Gibbs Free Energy= 0.399259
SCF energy+Solvation free energy (Methanol)= -2078.72305720
SCF energy+Solvation free energy (Ethanol)= -2078.72228074

Coordinates:

6 -1.87445 -0.292515 2.05598
1 -2.10998 -1.11564 2.74531
6 -0.73122 0.539827 2.65978
1 -0.651968 1.52849 2.19984
1 -0.709522 0.631596 3.74562
6 0.21738 -0.486049 2.01763
1 0.408244 -1.31936 2.70676
6 1.5094 -0.069379 1.35374
1 1.99217 -0.954236 0.926512
1 2.19801 0.37166 2.08344
6 -1.04013 -2.30866 0.745455
1 -1.26836 -2.83402 1.68396
1 -1.8973 -2.38043 0.069472
6 0.218534 -2.86841 0.131979
6 1.16939 -3.50031 0.936611
1 0.950684 -3.67915 1.98759
6 2.39666 -3.89753 0.408928
1 3.12845 -4.38513 1.04568
6 2.67654 -3.67241 -0.937266
1 3.63151 -3.98007 -1.35258
6 1.71671 -3.07691 -1.75607
1 1.91999 -2.92131 -2.81102
6 0.490265 -2.68132 -1.22802
1 -0.2598 -2.21586 -1.86343
6 2.4038 0.862895 -0.734552
1 2.14184 1.56721 -1.52584
1 2.43232 -0.140621 -1.17135
6 3.7091 1.21164 -0.0658
6 4.62631 0.217443 0.28015
1 4.40796 -0.818965 0.029284
6 5.81203 0.545004 0.932877
1 6.52065 -0.235683 1.19163
6 6.08922 1.87257 1.24896
1 7.01371 2.12931 1.75643
6 5.18176 2.87235 0.904264
1 5.40028 3.90984 1.13712
6 3.99988 2.54238 0.248153
1 3.30187 3.32574 -0.041622
6 -3.15349 0.302035 1.55479
6 -4.3084 -0.480699 1.58699
1 -4.26044 -1.48757 1.99654
6 -5.50985 0.008284 1.0862
1 -6.39878 -0.613949 1.10479
6 -5.56442 1.29374 0.556117
1 -6.49801 1.67769 0.157172
6 -4.41955 2.08673 0.533322
1 -4.45669 3.08552 0.111205
6 -3.21946 1.59426 1.03215
1 -2.32692 2.21096 0.978579
7 -0.876877 -0.863614 1.05348

7 1.25753 0.866249 0.228452
1 1.16885 1.81791 0.591008
17 -2.54851 -0.470852 -1.67441
17 -0.216258 1.86636 -2.4261
78 -0.587412 0.351207 -0.667452

4a second lowest energy N-benzyl rotamer

SCF energy= -2078.68444706
Zero-point correction= 0.458799
Thermal correction to Energy= 0.485347
Thermal correction to Enthalpy= 0.486291
Thermal correction to Gibbs Free Energy= 0.399277
SCF energy+Solvation free energy (Methanol)= -2078.72305894
SCF energy+Solvation free energy (Ethanol)= -2078.72228250

Coordinates:

6 1.87463 -0.293144 -2.05584
1 2.11004 -1.1165 -2.74493
6 0.73151 0.53922 -2.65982
1 0.652389 1.52801 -2.2001
1 0.70985 0.630757 -3.74569
6 -0.217228 -0.486408 -2.01752
1 -0.408097 -1.31986 -2.70646
6 -1.50926 -0.069503 -1.3538
1 -1.99213 -0.954263 -0.926469
1 -2.19778 0.371461 -2.08363
6 1.03994 -2.3088 -0.744747
1 1.26833 -2.83445 -1.68304
1 1.89694 -2.38047 -0.068539
6 -0.218918 -2.86823 -0.131378
6 -1.16959 -3.50043 -0.936002
1 -0.950658 -3.67964 -1.98687
6 -2.39699 -3.89744 -0.408461
1 -3.12864 -4.38527 -1.04522
6 -2.6772 -3.6718 0.937577
1 -3.63226 -3.97932 1.35279
6 -1.71756 -3.07597 1.75638
1 -1.92109 -2.91996 2.81122
6 -0.490978 -2.68061 1.22848
1 0.258918 -2.21488 1.86389
6 -2.40376 0.86308 0.734347
1 -2.14183 1.56751 1.52554
1 -2.43228 -0.140366 1.17131
6 -3.70908 1.21165 0.065529
6 -4.62623 0.217312 -0.280145
1 -4.40777 -0.819034 -0.029099
6 -5.81203 0.544635 -0.932874
1 -6.52062 -0.236157 -1.19141
6 -6.08932 1.87211 -1.24923
1 -7.01388 2.12868 -1.75669
6 -5.18191 2.87204 -0.904819
1 -5.40053 3.90945 -1.13791
6 -3.99996 2.54231 -0.248717
1 -3.30199 3.32579 0.040839

6 3.15372 0.301374 -1.55476
6 4.30851 -0.481575 -1.58658
1 4.26041 -1.4886 -1.99574
6 5.51001 0.007402 -1.0859
1 6.39883 -0.614997 -1.10418
6 5.56476 1.29307 -0.556321
1 6.49839 1.67702 -0.157475
6 4.42002 2.08626 -0.533921
1 4.45729 3.08522 -0.112211
6 3.21988 1.5938 -1.03264
1 2.32743 2.21067 -0.979406
7 0.876919 -0.863824 -1.05319
7 -1.25743 0.866286 -0.228609
1 -1.16871 1.8179 -0.591298
17 2.54852 -0.470496 1.67459
17 0.216303 1.86697 2.42572
78 0.587477 0.351436 0.667373

4a highest energy N-benzyl rotamer

SCF energy= -2078.68588399
Zero-point correction= 0.459351
Thermal correction to Energy= 0.485599
Thermal correction to Enthalpy= 0.486543
Thermal correction to Gibbs Free Energy= 0.401722
SCF energy+Solvation free energy (Methanol)= -2078.72425858
SCF energy+Solvation free energy (Ethanol)= -2078.72345258

Coordinates:

6 2.20874 -1.56426 -1.16226
1 2.37742 -2.64948 -1.14852
6 1.58819 -1.18197 -2.51545
1 1.71862 -0.120953 -2.7445
1 1.88668 -1.77947 -3.37675
6 0.226008 -1.50255 -1.87379
1 -0.052154 -2.5501 -2.04135
6 -0.9737 -0.607581 -2.09381
1 -1.7937 -0.939819 -1.44997
1 -1.30638 -0.650892 -3.14064
6 0.487572 -2.36392 0.511197
1 0.80917 -3.34511 0.133667
1 1.06626 -2.11232 1.4045
6 -0.99307 -2.38202 0.781578
6 -1.81759 -3.28408 0.10467
1 -1.37508 -4.02148 -0.562559
6 -3.19875 -3.25368 0.283608
1 -3.83082 -3.95708 -0.249799
6 -3.76128 -2.32449 1.15461
1 -4.83753 -2.2909 1.29443
6 -2.94132 -1.4434 1.85663
1 -3.3764 -0.730381 2.54826
6 -1.56286 -1.46663 1.67161
1 -0.918204 -0.778024 2.21307
6 -1.82811 1.69446 -1.77266
1 -2.16329 1.71273 -2.81856

1 -1.45351 2.68229 -1.49946
6 -2.98962 1.31343 -0.887398
6 -3.09366 1.82273 0.410738
1 -2.28419 2.42783 0.810479
6 -4.23465 1.57186 1.16847
1 -4.31496 1.98973 2.1676
6 -5.27031 0.797606 0.650585
1 -6.16052 0.608829 1.24381
6 -5.163 0.267201 -0.632508
1 -5.96533 -0.337359 -1.04458
6 -4.03145 0.533595 -1.39734
1 -3.96639 0.150554 -2.41424
6 3.41004 -0.881487 -0.583968
6 4.25502 -1.61509 0.249447
1 4.04009 -2.66464 0.438625
6 5.35537 -1.01418 0.851236
1 5.99809 -1.59422 1.50563
6 5.62211 0.33121 0.616149
1 6.47444 0.807637 1.09006
6 4.79097 1.06813 -0.224019
1 4.98845 2.12007 -0.40159
6 3.69154 0.464913 -0.822929
1 3.03564 1.06326 -1.449
7 0.845838 -1.34498 -0.510545
7 -0.642825 0.781489 -1.7071
1 0.015017 1.15928 -2.39149
17 1.68144 0.383567 2.08419
17 0.24219 2.98376 0.470802
78 0.494982 0.67463 0.075706

4b lowest energy N-benzyl rotamer

SCF energy= -2078.68334346
Zero-point correction= 0.459258
Thermal correction to Energy= 0.485796
Thermal correction to Enthalpy= 0.486740
Thermal correction to Gibbs Free Energy= 0.399768
SCF energy+Solvation free energy (Methanol)= -2078.72101694
SCF energy+Solvation free energy (Ethanol)= -2078.72025613

Coordinates:

6 1.77232 0.311008 2.04794
1 2.21981 -0.299958 2.84343
6 0.439955 0.891903 2.54692
1 0.368577 1.11136 3.6124
1 0.140777 1.76532 1.96792
6 -0.21191 -0.41378 2.05452
1 -0.224818 -1.16078 2.85892
6 -1.54471 -0.437286 1.32551
1 -2.34486 -0.046307 1.96579
1 -1.78519 -1.47355 1.06974
6 2.83894 1.15036 1.41463
6 2.53971 2.29157 0.667928
1 1.50836 2.60593 0.532174
6 3.55512 3.01316 0.052127

1 3.30857 3.88778 -0.54059
6 4.87998 2.60309 0.178858
1 5.67071 3.16191 -0.311617
6 5.18806 1.47145 0.927885
1 6.21827 1.14494 1.02699
6 4.16985 0.752813 1.54512
1 4.40883 -0.135953 2.1251
6 1.53269 -2.0306 1.11915
1 2.41052 -1.98531 0.468032
1 1.8484 -2.30111 2.13687
6 0.512518 -3.02347 0.623204
6 -0.268293 -3.73638 1.53649
1 -0.085739 -3.62318 2.60353
6 -1.27071 -4.59756 1.0951
1 -1.87029 -5.14656 1.81462
6 -1.49213 -4.75786 -0.270493
1 -2.27194 -5.42778 -0.61938
6 -0.697821 -4.07244 -1.1885
1 -0.854228 -4.20882 -2.25384
6 0.30388 -3.21212 -0.747234
1 0.926929 -2.67821 -1.46098
6 -2.00036 1.72831 0.129293
1 -1.52583 2.23289 0.97284
1 -1.68467 2.21684 -0.795937
6 -3.50136 1.73369 0.266119
6 -4.1261 2.01031 1.48254
1 -3.5225 2.27452 2.34837
6 -5.51456 1.9645 1.59013
1 -5.99182 2.18424 2.53999
6 -6.28666 1.63888 0.478823
1 -7.36838 1.60299 0.560913
6 -5.67013 1.36617 -0.741731
1 -6.27036 1.12266 -1.61255
6 -4.2845 1.41591 -0.848339
1 -3.79751 1.22248 -1.80322
17 -0.451011 0.874077 -2.78235
17 2.57235 -0.364317 -1.65181
7 0.984984 -0.648122 1.16529
7 -1.46833 0.332447 0.056121
78 0.445539 0.093219 -0.747351
1 -2.04568 -0.143377 -0.641373

4b second lowest energy N-benzyl rotamer

SCF energy= -2078.67633560
Zero-point correction= 0.458600
Thermal correction to Energy= 0.485404
Thermal correction to Enthalpy= 0.486348
Thermal correction to Gibbs Free Energy= 0.398129
SCF energy+Solvation free energy (Methanol)= -2078.71629588
SCF energy+Solvation free energy (Ethanol)= -2078.71553863

Coordinates:

6 -1.62966 -0.37036 -1.30954
1 -2.41485 -1.12821 -1.41618

6 -0.345721 -0.887525 -1.97911
1 -0.470353 -1.52563 -2.85434
1 0.338886 -0.069621 -2.20488
6 -0.047092 -1.5855 -0.641583
1 -0.498131 -2.58473 -0.645536
6 1.33814 -1.65826 -0.027926
1 2.0197 -2.23284 -0.666699
1 1.26873 -2.17432 0.935798
6 -2.19868 0.979306 -1.62843
6 -1.38514 2.09063 -1.861
1 -0.304324 2.00401 -1.78386
6 -1.94982 3.32694 -2.14744
1 -1.30724 4.18679 -2.30583
6 -3.33445 3.46564 -2.2138
1 -3.77364 4.43452 -2.43001
6 -4.15303 2.36137 -2.00046
1 -5.23238 2.4614 -2.05652
6 -3.58384 1.12465 -1.71282
1 -4.22217 0.257474 -1.55923
6 -1.84213 -1.10643 1.1543
1 -1.17233 -1.32147 1.99351
1 -2.45322 -0.247093 1.44409
6 -2.69173 -2.31059 0.833329
6 -4.01284 -2.15184 0.404741
1 -4.4249 -1.14866 0.323688
6 -4.80218 -3.25892 0.105746
1 -5.82722 -3.1191 -0.22288
6 -4.28036 -4.54357 0.23933
1 -4.89514 -5.40765 0.008225
6 -2.97262 -4.71678 0.686042
1 -2.56718 -5.71564 0.812939
6 -2.18856 -3.60616 0.985715
1 -1.17748 -3.74522 1.36437
6 2.78415 0.243336 -0.818134
1 2.25972 0.205004 -1.77405
1 2.94936 1.29047 -0.551748
6 4.08126 -0.521545 -0.879172
6 4.33568 -1.4433 -1.8955
1 3.60288 -1.58339 -2.68747
6 5.52308 -2.17201 -1.90847
1 5.71266 -2.8842 -2.70536
6 6.46399 -1.98364 -0.900333
1 7.38961 -2.55029 -0.908615
6 6.22042 -1.06015 0.115694
1 6.95877 -0.902268 0.895317
6 5.03602 -0.330985 0.124924
1 4.85027 0.405888 0.905056
17 1.90776 2.39001 1.6858
17 -1.53581 2.20833 1.56231
7 -0.972446 -0.610737 0.043152
7 1.87776 -0.30073 0.238875
78 0.283285 0.927343 0.813486
1 2.4441 -0.335449 1.08963

4b highest energy N-benzyl rotamer

SCF energy= -2078.67637708

Zero-point correction= 0.458584
Thermal correction to Energy= 0.485365
Thermal correction to Enthalpy= 0.486309
Thermal correction to Gibbs Free Energy= 0.397732
SCF energy+Solvation free energy (Methanol)= -2078.71580592
SCF energy+Solvation free energy (Ethanol)= -2078.71504319

Coordinates:

6 -1.72126 0.280766 1.29924
1 -2.53171 1.01093 1.412
6 -0.480043 0.803102 2.04115
1 -0.65417 1.37601 2.95216
1 0.232387 -0.001909 2.22824
6 -0.173531 1.59407 0.757743
1 -0.677744 2.56675 0.797823
6 1.22663 1.7642 0.198616
1 1.85416 2.35741 0.875214
1 1.16226 2.30067 -0.754276
6 -2.26451 -1.09736 1.52776
6 -1.43358 -2.19945 1.74162
1 -0.353172 -2.08432 1.71594
6 -1.97858 -3.46128 1.94419
1 -1.32113 -4.31246 2.08739
6 -3.3608 -3.63539 1.94493
1 -3.78403 -4.62358 2.09528
6 -4.19755 -2.54119 1.7511
1 -5.27539 -2.66872 1.75692
6 -3.64805 -1.27897 1.54756
1 -4.30154 -0.420297 1.41107
6 -1.86218 1.11108 -1.14161
1 -1.16715 1.39342 -1.93943
1 -2.41635 0.236007 -1.49338
6 -2.78398 2.2578 -0.81052
6 -4.11037 2.01566 -0.442004
1 -4.47212 0.990361 -0.418583
6 -4.96672 3.06816 -0.130892
1 -5.99531 2.86337 0.148962
6 -4.50709 4.38174 -0.193247
1 -5.1742 5.20405 0.045088
6 -3.19413 4.6383 -0.580122
1 -2.8368 5.66082 -0.651318
6 -2.3423 3.58186 -0.890927
1 -1.32499 3.78706 -1.21981
6 2.7403 -0.061817 1.01411
1 2.25876 0.137043 1.97373
1 2.81873 -1.14199 0.870025
6 4.09609 0.591645 0.933721
6 4.47985 1.61306 1.80279
1 3.80867 1.92216 2.60144
6 5.72443 2.22522 1.6637
1 6.01789 3.01648 2.34647
6 6.59026 1.81827 0.65282
1 7.56044 2.29295 0.544939
6 6.21442 0.794989 -0.21652
1 6.89215 0.467997 -0.998676
6 4.97383 0.183079 -0.076423

1 4.6756 -0.625097 -0.743433
17 2.07898 -2.22135 -1.5512
17 -1.37034 -2.17042 -1.66647
7 -1.02071 0.611122 -0.011991
7 1.84401 0.442859 -0.074104
78 0.341363 -0.841444 -0.762482
1 2.44171 0.524596 -0.899935

Computational and crystallographically determined bond lengths, angles and torsion comparison

		**4a** / XRD (average)	**4a** / computational	**4b** / XRD (average)	**4b** / computational
**Pt–N(azet) (Å)**	Pt–N(azet)	2.086	2.132	2.062	2.121
**Pt–N(amin) (Å)**	Pt–N(amin)	2.058	2.118	2.052	2.089
**N–Pt–N (°)**	N–Pt–N	85.4	84.53	86.59	85.77
**N–C–C–N (°)**	N–C–C–N	-44.4	-49.42	45.2	44.28

Single crystal X-ray diffraction information

For crystals of **2a**, **2c**-**2g**, **3**, **4b**, **7a**, **7b** and **9:** The datasets were measured on an Agilent SuperNova diffractometer using an Atlas detector. The data collections were driven and processed and absorption corrections were applied using CrysAlisPro.[2] The structures of **2a**, **2c**, **2d**, **2g**, **3**, **4b** and **9** were solved using ShelXS[3] while **2e**, **2f**, **7a** and **7b** were solved using Superflip.[4] All structures were refined by a full-matrix least-squares procedure on F^2 in ShelXL.[5] All non-hydrogen atoms were refined with anisotropic displacement parameters. All hydrogen atoms were added at calculated positions and refined by use of a riding model with isotropic displacement parameters based on the equivalent isotropic displacement parameter (U_{eq}) of the parent atom, unless otherwise mentioned herein. Reports were produced using OLEX2.[6]

For crystals of **2b**, **4a** and **5**: The datasets were measured by the EPSRC UK National Crystallography Service[7] on a Bruker-Nonius Roper CCD diffractometer for **2b** and **4a** and on a Bruker-Nonius APEX II CCD diffractometer for **5**, all at the window of a Bruker-Nonius FR591 rotating anode. The data collections were driven by COLLECT[8] and processed by DENZO[9] and absorption corrections were applied using SADABS.[10] The structure of **5** was solved using ShelXS-97,[3] that of **4a** was solved in Sir2004,[11] and that of

2b was solved in Sir92.[12] All three structures were refined by a full-matrix least-squares procedure on F^2 in SHELXL.[5] All non-hydrogen atoms were refined with anisotropic displacement parameters, apart from those of the diethyl ether molecule in **2b**. All hydrogen atoms were added at calculated positions and refined by use of a riding model with isotropic displacement parameters based on the equivalent isotropic displacement parameter (U_{eq}) of the parent atom, unless otherwise mentioned herein. Reports were produced using OLEX2.[6]

The CIFs for **4a**, **2f**, **2d**, **2e**, **2c**, **2g**, **4b**, **2b**, **7b**, **3**, **5**, **9**, **2a** and **7a** have been deposited with the CCDC and have been given the deposition numbers CCDC 1824097, CCDC 1833714 - CCDC 1833719 and CCDC 1833721 - CCDC 1833727 respectively. These numbers contain the supplementary crystallographic data for this paper. These data can be obtained free of charge from The Cambridge Crystallographic Data Centre *via* www.ccdc.cam.ac.uk/data_request/cif.

Crystal structure determination of 2a

Crystal Data for $C_{26}H_{29}Cl_2N_3Pd$ (M =560.82 g/mol): monoclinic, space group P2$_1$ (no. 4), a = 10.69270(10) Å, b = 10.41080(10) Å, c = 43.8600(4) Å, β = 93.8860(10)°, V = 4871.25(8) Å^3, Z = 8, T = 100.00(10) K, μ(CuKα) = 8.303 mm^{-1}, $Dcalc$ = 1.529 g/cm^3, 85740 reflections measured (6.06° ≤ 2Θ ≤ 133.198°), 17235 unique (R_{int} = 0.0493, R_{sigma} = 0.0338) which were used in all calculations. The final R_1 was 0.0473 (I > 2σ(I)) and wR_2 was 0.1155 (all data). Flack = -0.012 (5). **CCDC 1833726**.

The crystal structure of **2a** contains four crystallographically-independent palladium complexes with four molecules of acetonitrile. The absolute structure has been determined from the diffraction data. The hydrogen atoms bonded to N(2), N(102), N(202) and N(302) (one per crystallographically-independent palladium complex) were located in the electron density and their positions refined subject to suitable distance restraints, (N-H = 0.9 (2) Å). The isotropic thermal parameters (U_{iso}) of these hydrogen atoms were set at 1.2 times the equivalent isotropic thermal parameter (U_{eq}) of the parent nitrogen atom.

Crystal structure determination of 2b

Crystal Data for $C_{25.4}H_{29}Cl_2N_2O_{1.1}Pd$ (M =557.20 g/mol): triclinic, space group P-1 (no. 2), a = 13.4719(2) Å, b = 19.3528(2) Å, c = 21.6914(3) Å, α = 94.4120(10)°, β = 106.4250(10)°, γ = 108.6670(10)°, V = 5051.22(12) Å^3, Z = 8, T = 120 (2) K, μ(MoKα) = 0.966 mm^{-1}, $Dcalc$ = 1.465 g/cm^3, 105521 reflections measured (6.002° ≤ 2Θ ≤ 55.098°), 23199 unique (R_{int} = 0.0436,

R_{sigma} = 0.0413) which were used in all calculations. The final R_1 was 0.0377 (I > 2σ(I)) and wR_2 was 0.0910 (all data). **CCDC 1833721.**

The crystal structure of **2b** contains four crystallographically-independent palladium complexes with 1/10th of a diethyl ether molecule per palladium complex (0.4 per asymmetric unit). It was not possible to refine the atoms of the diethyl ether molecule anisotropically. The hydrogen atoms bonded to N(2), N(102), N(202) and N(302) (one per crystallographically-independent palladium complex) were located in the electron density and their positions refined subject to suitable distance and angle restraints, (N-H = 0.91 (2) Å and Pd … H = 2.48 (4) Å). The isotropic thermal parameters (U_{iso}) of these hydrogen atoms were set at 1.2 times the equivalent isotropic thermal parameter (U_{eq}) of the parent nitrogen atom.

Crystal structure determination of 2c

Crystal Data for $C_{20}H_{26}Cl_2N_2Pd$ (M =471.73 g/mol): monoclinic, space group $P2_1/c$ (no. 14), a = 10.25753(11) Å, b = 12.98993(13) Å, c = 14.97010(16) Å, β = 97.8307(10)°, V = 1976.08(4) Å^3, Z = 4, T = 100.00(10) K, μ(CuKα) = 10.089 mm^{-1}, $Dcalc$ = 1.586 g/cm^3, 18864 reflections measured (13.122° ≤ 2Θ ≤ 140.144°), 3745 unique (R_{int} = 0.0279, R_{sigma} = 0.0193) which were used in all calculations. The final R_1 was 0.0202 (I > 2σ(I)) and wR_2 was 0.0487 (all data). **CCDC 1833717.**

The hydrogen atom bonded to N(2) was located in the electron density and the position refined.

Crystal structure determination of 2d

Crystal Data for $C_{21}H_{28}Cl_2N_2OPd$ (M =501.75 g/mol): orthorhombic, space group Pbca (no. 61), a = 13.1563(3) Å, b = 15.1527(3) Å, c = 20.3207(4) Å, V = 4050.99(15) Å^3, Z = 8, T = 99.98(10) K, μ(MoKα) = 1.194 mm^{-1}, $Dcalc$ = 1.645 g/cm^3, 15417 reflections measured (6.194° ≤ 2Θ ≤ 52.744°), 4143 unique (R_{int} = 0.0307, R_{sigma} = 0.0297) which were used in all calculations. The final R_1 was 0.0244 (I > 2σ(I)) and wR_2 was 0.0571 (all data). **CCDC 1833715.**

Crystal structure determination of 2e

Crystal Data for $C_{21}H_{26}Cl_2N_2Pd$ (M =483.74 g/mol): monoclinic, space group $P2_1/c$ (no. 14), a = 17.4908(3) Å, b = 7.87326(8) Å, c = 15.9571(2) Å, β = 112.3221(16)°, V = 2032.78(5) Å^3, Z = 4, T = 100.01(10) K, μ(CuKα) = 9.825 mm^{-1}, $Dcalc$ = 1.581 g/cm^3, 24316 reflections measured (12.742° ≤ 2Θ ≤ 148.964°), 3993 unique (R_{int} = 0.0316, R_{sigma} = 0.0171) which were used in all calculations. The final R_1 was 0.0196 (I > 2σ(I)) and wR_2 was 0.0525 (all data). **CCDC 1833716.**

Crystal structure determination of 2f

Crystal Data for $C_{22}H_{28}Cl_2N_2OPd$ (M =513.76 g/mol): monoclinic, space group $P2_1/c$ (no. 14), a = 7.80137(15) Å, b = 16.1647(4) Å, c = 16.8824(4) Å, β = 90.108(2)°, V = 2128.98(9) Å^3, Z = 4, T = 100.00(10) K, μ(MoKα) = 1.138 mm^{-1}, $Dcalc$ = 1.603 g/cm^3, 12063 reflections measured (6.278° ≤ 2Θ ≤ 52.744°), 4357 unique (R_{int} = 0.0266, R_{sigma} = 0.0350) which were used in all calculations. The final R_1 was 0.0255 (I > 2σ(I)) and wR_2 was 0.0550 (all data). **CCDC 1833714**.

Crystal structure determination of 2g

Crystal Data for $C_{27}H_{31}Cl_2N_3Pd$ (M =574.85 g/mol): orthorhombic, space group $Pna2_1$ (no. 33), a = 20.32152(15) Å, b = 13.97093(7) Å, c = 18.13038(9) Å, V = 5147.40(5) Å^3, Z = 8, T = 100.00(10) K, μ(CuKα) = 7.871 mm^{-1}, $Dcalc$ = 1.484 g/cm^3, 46752 reflections measured (13.088° ≤ 2Θ ≤ 148.916°), 10128 unique (R_{int} = 0.0268, R_{sigma} = 0.0193) which were used in all calculations. The final R_1 was 0.0172 (I > 2σ(I)) and wR_2 was 0.0427 (all data). Flack = -0.028 (2). **CCDC 1833718**.

The crystal structure of **2g** contains two crystallographically-independent palladium complexes with two molecules of acetonitrile. The absolute structure has been determined from the diffraction data.

Crystal structure determination of 3

Crystal Data for $C_{28.5}H_{35}ClN_2O_5Pd$ (M =627.43 g/mol): monoclinic, space group C2/c (no. 15), a = 20.8287(2) Å, b = 15.6540(2) Å, c = 17.0460(2) Å, β = 93.4250(10)°, V = 5547.96(11) Å^3, Z = 8, T = 100.00(10) K, μ(CuKα) = 6.617 mm^{-1}, $Dcalc$ = 1.502 g/cm^3, 49813 reflections measured (7.068° ≤ 2Θ ≤ 140.118°), 5263 unique (R_{int} = 0.0479, R_{sigma} = 0.0204) which were used in all calculations. The final R_1 was 0.0252 (I > 2σ(I)) and wR_2 was 0.0683 (all data). **CCDC 1833723**.

The crystal structure of **3** includes a dichloromethane molecule which lies on a two-fold rotation axis such that only half the molecule is unique. The hydrogen atoms bonded to O(3) and N(2) were located in the electron density and their positions refined. The isotropic thermal parameters (U_{iso}) of these hydrogen atoms were set at 1.5 times and 1.2 times the equivalent isotropic thermal parameter (U_{eq}) of the parent oxygen atom and parent nitrogen atom respectively.

Crystal structure determination of 4a.

Crystal Data for $C_{26}H_{29}Cl_2N_3Pt$ (M =649.51 g/mol):[13, 14] monoclinic, space group $P2_1$ (no. 4), a = 10.6958(2) Å, b = 10.4600(2) Å, c = 43.9267(10) Å, β = 93.9630(10)°, V = 4902.68(17) Å^3, Z = 8, T = 120 (2) K, μ(MoKα) = 5.961 mm^{-1}, $Dcalc$ = 1.760 g/cm^3, 44832 reflections measured (5.84° ≤ 2Θ ≤ 50.06°),

16268 unique (R_{int} = 0.1064) which were used in all calculations. The final R_1 was 0.0623 (>2σ(I)) and wR_2 was 0.0908 (all data). Flack = 0.041 (7). **CCDC 1824097**.

This structure has been previously reported by us,[14] and is discussed in this report and re-presented for completeness of the discussion herein. The structure contains four crystallographically-independent platinum complexes with one acetonitrile molecule per platinum complex. The absolute structure has been determined from the diffraction data. The hydrogen atoms bonded to N(2), N(102), N(202) and N(302) (one per crystallographically-independent platinum complex) were located in the electron density and their positions refined subject to suitable distance and angle restraints, (N-H = 0.91 (2) Å, Pd … H = 2.48 (4) Å and C(104) … H(102) = C(112) … H(102) = 1.92 (4) Å). The isotropic thermal parameters (U_{iso}) of these hydrogen atoms were set at 1.2 times the equivalent isotropic thermal parameter (U_{eq}) of the parent nitrogen atom.

Crystal structure determination of 4b

Crystal Data for $C_{24}H_{26}Cl_2N_2Pt$ (M =608.46 g/mol): monoclinic, space group $P2_1/c$ (no. 14), a = 10.57037(14) Å, b = 16.66192(19) Å, c = 12.69181(17) Å, β = 102.0822(13)°, V = 2185.80(5) Å^3, Z = 4, T = 100.00(11) K, μ(CuKα) = 14.347 mm^{-1}, $Dcalc$ = 1.849 g/cm^3, 16242 reflections measured (12.798° ≤ 2Θ ≤ 140.104°), 4130 unique (R_{int} = 0.0327, R_{sigma} = 0.0261) which were used in all calculations. The final R_1 was 0.0200 (I > 2σ(I)) and wR_2 was 0.0498 (all data). **CCDC 1833719**.

The hydrogen atom bonded to N(2) was located in the electron density and the position refined.

Crystal structure determination of 5

Crystal Data for $C_{28}H_{33.76375}BrN_2O_{1.6175}Pd$ (M =610.52 g/mol): monoclinic, space group C2/c (no. 15), a = 30.2910(5) Å, b = 9.6252(2) Å, c = 17.6711(4) Å, β = 90.9440(10)°, V = 5151.43(18) Å^3, Z = 8, T = 120 (2) K, μ(MoKα) = 2.298 mm^{-1}, $Dcalc$ = 1.574 g/cm^3, 26372 reflections measured (5.848° ≤ 2Θ ≤ 53.462°), 5459 unique (R_{int} = 0.0349, R_{sigma} = 0.0295) which were used in all calculations. The final R_1 was 0.0273 (I > 2σ(I)) and wR_2 was 0.0615 (all data). **CCDC 1833724**.

The crystal structure of **5** includes a molecule of solvent disordered such that O(101), O(102), C(101)-C(104) is an ethyl acetate molecule while O(11'), C(11')-C(14') is an ether molecule, with a refined percentage occupancy ratio of 61.6(11):38.4(11) respectively. The hydrogen atom bonded to N(2) was located in the electron density and the position refined.

Crystal structure determination of 7a

Crystal Data for $C_{28}H_{35}Cl_2N_3OPd$ (M =606.89 g/mol): monoclinic, space group C2/c (no. 15), a = 19.6145(5) Å, b = 8.5482(2) Å, c = 32.4998(9) Å, β = 95.603(3)°, V = 5423.2(3) Å^3, Z = 8, T = 100.00(10) K, μ(MoKα) = 0.907 mm^{-1}, $Dcalc$ = 1.487 g/cm^3, 10858 reflections measured (5.868° ≤ 2Θ ≤ 50.7°), 4960 unique (R_{int} = 0.0250, R_{sigma} = 0.0397) which were used in all calculations. The final R_1 was 0.0391 (I > 2σ(I)) and wR_2 was 0.0719 (all data). **CCDC 1833727**.

The crystal structure of **7a** includes one molecule of acetonitrile per palladium complex. The atoms C(24A)-C(25A) / C(24B)-C(25B) are disordered over two positions with a refined percentage occupancy ratio of 56.1(6):43.9(6) respectively.

Crystal structure determination of 7b

Crystal Data for $C_{27}H_{34}Cl_2N_2OPd$ (M =579.86 g/mol): triclinic, space group P-1 (no. 2), a = 8.6263(3) Å, b = 12.0854(5) Å, c = 12.8671(7) Å, α = 77.377(4)°, β = 75.472(4)°, γ = 84.423(3)°, V = 1265.87(11) Å^3, Z = 2, T = 100.00(10) K, μ(CuKα) = 8.023 mm^{-1}, $Dcalc$ = 1.521 g/cm^3, 8744 reflections measured (7.246° ≤ 2Θ ≤ 140.148°), 4775 unique (R_{int} = 0.0303, R_{sigma} = 0.0425) which were used in all calculations. The final R_1 was 0.0334 (I > 2σ(I)) and wR_2 was 0.0890 (all data). **CCDC 1833722**.

The hydrogen atom bonded to N(1) was located in the electron density and the position refined subject to a suitable distance restraint, (N-H = 0.91 (2) Å).

Crystal structure determination of 9

Crystal Data for $C_{21}H_{28}Cl_2N_2OPd$ (M =501.75 g/mol): monoclinic, space group $P2_1/n$ (no. 14), a = 11.8735(2) Å, b = 13.8678(3) Å, c = 12.9692(3) Å, β = 102.406(2)°, V = 2085.65(7) Å^3, Z = 4, T = 100.01(10) K, μ(MoKα) = 1.160 mm^{-1}, $Dcalc$ = 1.598 g/cm^3, 11320 reflections measured (5.876° ≤ 2Θ ≤ 52.736°), 4264 unique (R_{int} = 0.0225, R_{sigma} = 0.0291) which were used in all calculations. The final R_1 was 0.0223 (I > 2σ(I)) and wR_2 was 0.0477 (all data). **CCDC 1833725**.

The hydrogen atom bonded to N(2) was located in the electron density and the position refined.

Further information added to clarify issues surrounding symmetry

Structure **2a** is in a chiral space group with a Flack parameter of 0. There are four molecules in the asymmetric unit – that is four crystallographically unique molecules, with nitrogen atoms labelled N(1) & N(2) for molecule A, N(101) & N(102) for molecule B, N(201) & N(202) for molecule C and N(301) and N(302)

for molecule D. There are two asymmetric units in the unit cell, so two examples each of molecules A, B, C and D, so 8 molecules in the unit cell. Thus, within the asymmetric unit there are a 50:50 mixture of enantiomers. However, there are eight molecules in the unit cell and the Flack parameter tells us that the symmetry related version of molecule A, for example, will also have N(1) & N(2) being of one stereogenicity, while the symmetry related version of molecule B will also have N(101) & N(102) being the opposite stereogenicity, etc.

Structures **2b-2f** are all in centrosymmetric space groups, thus there are inversion centres present.

Structure **2g** is different. It is a polar space group with a Flack parameter of 0. The space group is *Pna*2(1) which involves two glide planes. Glide planes combine a reflection operation with a translation, so the reflection part of that results in an inversion of symmetry.

Tables of selected bond lengths, angle and torsions from XRD analysis

		**2a**	**2b**	**2c**	**2d**	**2e**	**2f**	**2g***
**Pd–N(azet) (Å)**	Pd(1)–N(1)	2.088(8)	2.076(2)	2.0826(17)	2.0837(19)	2.0601(14)	2.0749(19)	2.074(2)
	Pd(11)–N(101)	2.094(8)	2.066(3)					2.072(2)
	Pd(21)–N(201)	2.069(11)	2.077(2)					
	Pd(31)–N(301)	2.127(10)	2.061(2)					
**Pd–N(amin) (Å)**	Pd(1)–N(2)	2.052(10)	2.058(2)	2.0756(17)	2.0791(19)	2.0794(14)	2.0696(18)	2.079(3)
	Pd(11)–N(102)	2.075(10)	2.065(3)					2.081(3)
	Pd(21)–N(202)	2.073(10)	2.075(2)					
	Pd(31)–N(302)	2.063(9)	2.061(2)					
**N–Pd–N (°)**	N(1)–Pd(1)–N(2)	85.5(4)	85.23(10)	85.62(6)	85.81(7)	86.17(5)	86.48(7)	86.49(10)
	N(101)–Pd(11)–N(102)	85.6(4)	85.90(11)					87.04(10)
	N(201)–Pd(21)–N(202)	86.0(4)	84.20(9)					
	N(301)–Pd(31)–N(302)	85.4(4)	85.58(10)					
**N–C–C–N**	N(1)–C(3)–C(4)–N(2)	44.7(12)	-48.0(3)	-45.9(2)	46.8(2)	39.4(2)	-41.5(3)	44.7(3)

**(°)**	N(101)–C(103)–C(104)–N(102)	-49.9(13)	-38.9(4)					44.8(3)
	N(201)–C(203)–C(204)–N(202)	46.6(12)	-48.3(3)					
	N(301)–C(303)–C(304)–N(302)	-44.8(13)	-35.3(4)					

* In structure **2g** the palladium atoms are labelled Pd(1) and Pd(10) (instead of Pd(11)).

		4a	4b
**Pt–N(azet)**	Pt(1)–N(1)	2.092(7)	2.062(2)
**(Å)**	Pt(11)–N(101)	2.088(9)	
	Pt(21)–N(201)	2.075(8)	
	Pt(31)–N(301)	2.087(10)	
**Pt–N(amin)**	Pt(1)–N(2)	2.062(9)	2.052(2)
**(Å)**	Pt(11)–N(102)	2.060(9)	
	Pt(21)–N(202)	2.047(9)	
	Pt(31)–N(302)	2.061(9)	
**N–Pt–N**	N(1)–Pt(1)–N(2)	85.4(3)	86.59(1)
**(°)**	N(101)–Pt(11)–N(102)	85.3(3)	
	N(201)–Pt(21)–N(202)	85.3(3)	
	N(301)–Pt(31)–N(302)	85.6(3)	
**N–C–C–N**	N(1)–C(3)–C(4)–N(2)	-46.9(11)	45.2(3)
**(°)**	N(101)–C(103)–C(104)–N(102)	-46.8(12)	
	N(201)–C(203)–C(204)–N(202)	43.3(11)	
	N(301)–C(303)–C(304)–N(302)	44.4(11)	

References

1. (a) L. Falivene, R. Credendino, A. Poater, A. Petta, L. Serra, R. Oliva, V. Scarano and L. Cavallo, *Organometallics*, 2016, **35**, 2286-2293; (b) A. Poater, B. Cosenza, A. Correa, S. Giudice, F. Ragone, V. Scarano and L. Cavallo, *Eur J Inorg Chem*, 2009, **2009**, 1759-1766.
2. CrysAlisPro, 2013, **Version 1.171.36.28**, Agilent Technologies.
3. G. M. Sheldrick, *Acta Crystallogr., Sect. A*, 2008, **A64**, 112-122.
4. (a) L. Palatinus and G. Chapuis, *J. Appl. Crystallogr.*, 2007, **40**, 786-790; (b) L. Palatinus and A. van der Lee, *J. Appl. Crystallogr.*, 2008, **41**, 975-984; (c) L. Palatinus, S. J. Prathapa and S. van Smaalen, *J. Appl. Crystallogr.*, 2012, **45**, 575-580.
5. G. M. Sheldrick, *Acta Crystallogr A*, 2015, **C71**, 3-8.
6. O. V. Dolomanov, L. J. Bourhis, R. J. Gildea, J. A. K. Howard and H. Puschmann, *J. Appl. Crystallogr.*, 2009, **42**, 339-341.
7. S. J. Coles and P. A. Gale, *Chem. Sci.*, 2012, **3**, 683-689.
8. R. W. W. Hooft, *COLLECT Data Collection Software*, 1998, Nonius, B.V., Delft, The Netherlands.

9. Z. Otwinowski and W. Minor, eds. C. W. Carter and R. M. Sweet, Academic Press, New York, 1997, vol. 276, pp. 307-326.

10. G. M. Sheldrick, *SADABS*, Bruker AXS Inc., Madison, Wisconsin, USA, 2007.

11. M. C. Burla, R. Caliandro, M. Camalli, B. Carrozzini, G. L. Cascarano, L. De Caro, G. Giacovazzo, G. Polidori and R. Spagna, *J. Appl. Crystallogr.*, 2005, **38**, 381-388.

12. A. Altomare, G. Cascarano, C. Giacovazzo, A. Guagliardi, M. C. Burla, G. Polidori and M. Camalli, *J. Appl. Crystallogr.*, 1994, **27**, 435-436.

13. Previously reported by us in the following citation(s) as (rac)-7.

14. (a) A. Yoshizawa, A. Feula, L. Male, A. G. Leach and J. S. Fossey, *Scientific Reports*, 2018, **8**, 6541; (b) A. Yoshizawa, A. Feula, L. Male, A. G. Leach and J. S. Fossey, *ChemRxiv*, 2018, DOI: 10.26434/chemrxiv.5887243.v5887241.
