# Supplementary material for: Palladium and Platinum 2,4-cis-amino Azetidine and Related Complexes
Source: Front Chem. 2018 Jun 21;6:211. doi: 10.3389/fchem.2018.00211 (PMC6021532; doi:10.3389/fchem.2018.00211)

checkCIF/PLATON report

Structure factors have been supplied for datablock(s) 2f

THIS REPORT IS FOR GUIDANCE ONLY. IF USED AS PART OF A REVIEW PROCEDURE FOR PUBLICATION, IT SHOULD NOT REPLACE THE EXPERTISE OF AN EXPERIENCED CRYSTALLOGRAPHIC REFEREE.

No syntax errors found.   CIF dictionary   Interpreting this report

Datablock: 2f

```
Bond precision:  C-C = 0.0034 A          Wavelength=0.71073

Cell:            a=7.80137(15)     b=16.1647(4)       c=16.8824(4)
                 alpha=90          beta=90.108(2)     gamma=90
Temperature:     100 K

                Calculated                 Reported
Volume          2128.98(8)                 2128.98(9)
Space group     P 21/c                     P 1 21/c 1
Hall group      -P 2ybc                    -P 2ybc
Moiety formula  C22 H28 Cl2 N2 O Pd        C22 H28 Cl2 N2 O Pd
Sum formula     C22 H28 Cl2 N2 O Pd        C22 H28 Cl2 N2 O Pd
Mr              513.76                     513.76
Dx,g cm-3       1.603                      1.603
Z               4                          4
Mu (mm-1)       1.138                      1.138
F000            1048.0                     1048.0
F000'           1045.47
h,k,lmax        9,20,21                    9,20,21
Nref            4364                       4357
Tmin,Tmax       0.771,0.853                0.831,0.895
Tmin'           0.709

Correction method= # Reported T Limits: Tmin=0.831 Tmax=0.895
AbsCorr = GAUSSIAN

Data completeness= 0.998          Theta(max)= 26.372

R(reflections)= 0.0255( 3701)     wR2(reflections)= 0.0550( 4357)

S = 1.059                  Npar= 254
```

The following ALERTS were generated. Each ALERT has the format
test-name_ALERT_alert-type_alert-level.
Click on the hyperlinks for more details of the test.

Alert level C

```
PLAT910_ALERT_3_C Missing # of FCF Reflection(s) Below Theta(Min).         7 Note
```

Alert level G

```
PLAT793_ALERT_4_G Model has Chirality at N1         (Centro SPGR)          S Verify
PLAT793_ALERT_4_G Model has Chirality at C1         (Centro SPGR)          R Verify
PLAT793_ALERT_4_G Model has Chirality at C3         (Centro SPGR)          S Verify
PLAT978_ALERT_2_G Number C-C Bonds with Positive Residual Density.         8 Info
```

```
   0 ALERT level A = Most likely a serious problem - resolve or explain
   0 ALERT level B = A potentially serious problem, consider carefully
   1 ALERT level C = Check. Ensure it is not caused by an omission or oversight
   4 ALERT level G = General information/check it is not something unexpected

   0 ALERT type 1 CIF construction/syntax error, inconsistent or missing data
   1 ALERT type 2 Indicator that the structure model may be wrong or deficient
   1 ALERT type 3 Indicator that the structure quality may be low
   3 ALERT type 4 Improvement, methodology, query or suggestion
   0 ALERT type 5 Informative message, check
```

It is advisable to attempt to resolve as many as possible of the alerts in all categories. Often the minor alerts point to easily fixed oversights, errors and omissions in your CIF or refinement strategy, so attention to these fine details can be worthwhile. In order to resolve some of the more serious problems it may be necessary to carry out additional measurements or structure refinements. However, the purpose of your study may justify the reported deviations and the more serious of these should normally be commented upon in the discussion or experimental section of a paper or in the "special_details" fields of the CIF. checkCIF was carefully designed to identify outliers and unusual parameters, but every test has its limitations and alerts that are not important in a particular case may appear. Conversely, the absence of alerts does not guarantee there are no aspects of the results needing attention. It is up to the individual to critically assess their own results and, if necessary, seek expert advice.

Publication of your CIF in IUCr journals

A basic structural check has been run on your CIF. These basic checks will be run on all CIFs submitted for publication in IUCr journals (*Acta Crystallographica*, *Journal of Applied Crystallography*, *Journal of Synchrotron Radiation*); however, if you intend to submit to *Acta Crystallographica Section C* or *E* or *IUCrData*, you should make sure that full publication checks are run on the final version of your CIF prior to submission.

Publication of your CIF in other journals

Please refer to the *Notes for Authors* of the relevant journal for any special instructions relating to CIF submission.

PLATON version of 30/01/2018; check.def file version of 30/01/2018

Datablock 2f - ellipsoid plot

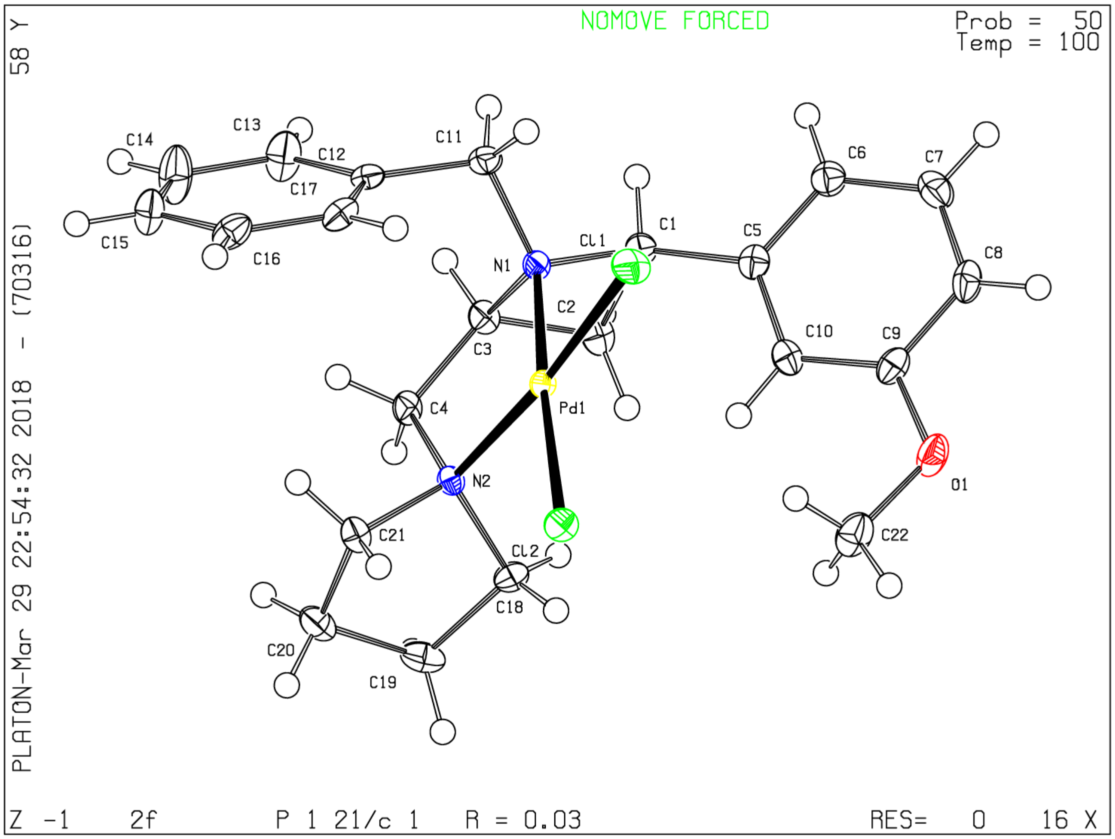

Supplement: Supplementary file 2 [file Data_Sheet_2.ZIP › cif checks/2f_CheckCIF.pdf]
