# Supplementary material for: Palladium and Platinum 2,4-cis-amino Azetidine and Related Complexes
Source: Front Chem. 2018 Jun 21;6:211. doi: 10.3389/fchem.2018.00211 (PMC6021532; doi:10.3389/fchem.2018.00211)

# checkCIF/PLATON report

Structure factors have been supplied for datablock(s) 2g

THIS REPORT IS FOR GUIDANCE ONLY. IF USED AS PART OF A REVIEW PROCEDURE FOR PUBLICATION, IT SHOULD NOT REPLACE THE EXPERTISE OF AN EXPERIENCED CRYSTALLOGRAPHIC REFEREE.

No syntax errors found.   CIF dictionary   Interpreting this report

## Datablock: 2g

---

```
Bond precision:  C-C = 0.0048 A          Wavelength=1.54184

Cell:           a=20.32152(15)     b=13.97093(7)     c=18.13038(9)
                alpha=90           beta=90           gamma=90
Temperature:    100 K

                Calculated                  Reported
Volume          5147.41(5)                  5147.40(5)
Space group     P n a 21                    P n a 21
Hall group      P 2c -2n                    P 2c -2n
Moiety formula  C25 H28 Cl2 N2 Pd, C2 H3 N  C25 H28 Cl2 N2 Pd, C2 H3 N
Sum formula     C27 H31 Cl2 N3 Pd           C27 H31 Cl2 N3 Pd
Mr              574.85                      574.85
Dx,g cm-3       1.484                       1.484
Z               8                           8
Mu (mm-1)       7.871                       7.871
F000            2352.0                      2352.0
F000'           2363.58
h,k,lmax        25,17,22                    25,17,22
Nref            10543[ 5445]                10128
Tmin,Tmax       0.348,0.467                 0.380,0.580
Tmin'           0.152

Correction method= # Reported T Limits: Tmin=0.380 Tmax=0.580
AbsCorr = GAUSSIAN

Data completeness= 1.86/0.96      Theta(max)= 74.458

R(reflections)= 0.0172( 9868)     wR2(reflections)= 0.0427( 10128)

S = 1.037                  Npar= 597
```

---

The following ALERTS were generated. Each ALERT has the format
**test-name_ALERT_alert-type_alert-level**.
Click on the hyperlinks for more details of the test.

## Alert level C

```
PLAT411_ALERT_2_C Short Inter H...H Contact  H10A     ..H17              2.00 Ang.
PLAT910_ALERT_3_C Missing # of FCF Reflection(s) Below Theta(Min).         10 Note
```

## Alert level G

```
PLAT142_ALERT_4_G s.u. on b - Axis Small or Missing .............    0.00007 Ang.
PLAT143_ALERT_4_G s.u. on c - Axis Small or Missing .............    0.00009 Ang.
PLAT232_ALERT_2_G Hirshfeld Test Diff (M-X) Pd1      --Cl2       .        5.3 s.u.
PLAT792_ALERT_1_G Model has Chirality at N1        (Polar  SPGR)            R Verify
PLAT792_ALERT_1_G Model has Chirality at N101      (Polar  SPGR)            R Verify
PLAT792_ALERT_1_G Model has Chirality at C1        (Polar  SPGR)            S Verify
PLAT792_ALERT_1_G Model has Chirality at C3        (Polar  SPGR)            R Verify
PLAT792_ALERT_1_G Model has Chirality at C101      (Polar  SPGR)            S Verify
PLAT792_ALERT_1_G Model has Chirality at C103      (Polar  SPGR)            R Verify
PLAT912_ALERT_4_G Missing # of FCF Reflections Above STh/L=  0.600         55 Note
PLAT913_ALERT_3_G Missing # of Very Strong Reflections in FCF ....          3 Note
PLAT978_ALERT_2_G Number C-C Bonds with Positive Residual Density.         15 Info
```

0 **ALERT level A** = Most likely a serious problem - resolve or explain
0 **ALERT level B** = A potentially serious problem, consider carefully
2 **ALERT level C** = Check. Ensure it is not caused by an omission or oversight
12 **ALERT level G** = General information/check it is not something unexpected

6 ALERT type 1 CIF construction/syntax error, inconsistent or missing data
3 ALERT type 2 Indicator that the structure model may be wrong or deficient
2 ALERT type 3 Indicator that the structure quality may be low
3 ALERT type 4 Improvement, methodology, query or suggestion
0 ALERT type 5 Informative message, check

It is advisable to attempt to resolve as many as possible of the alerts in all categories. Often the minor alerts point to easily fixed oversights, errors and omissions in your CIF or refinement strategy, so attention to these fine details can be worthwhile. In order to resolve some of the more serious problems it may be necessary to carry out additional measurements or structure refinements. However, the purpose of your study may justify the reported deviations and the more serious of these should normally be commented upon in the discussion or experimental section of a paper or in the "special_details" fields of the CIF. checkCIF was carefully designed to identify outliers and unusual parameters, but every test has its limitations and alerts that are not important in a particular case may appear. Conversely, the absence of alerts does not guarantee there are no aspects of the results needing attention. It is up to the individual to critically assess their own results and, if necessary, seek expert advice.

**Publication of your CIF in IUCr journals**

A basic structural check has been run on your CIF. These basic checks will be run on all CIFs submitted for publication in IUCr journals (*Acta Crystallographica*, *Journal of Applied Crystallography*, *Journal of Synchrotron Radiation*); however, if you intend to submit to *Acta Crystallographica Section C* or *E* or *IUCrData*, you should make sure that full publication checks are run on the final version of your CIF prior to submission.

**Publication of your CIF in other journals**

Please refer to the *Notes for Authors* of the relevant journal for any special instructions relating to CIF submission.

---

**PLATON version of 30/01/2018; check.def file version of 30/01/2018**

**Datablock 2g** - ellipsoid plot

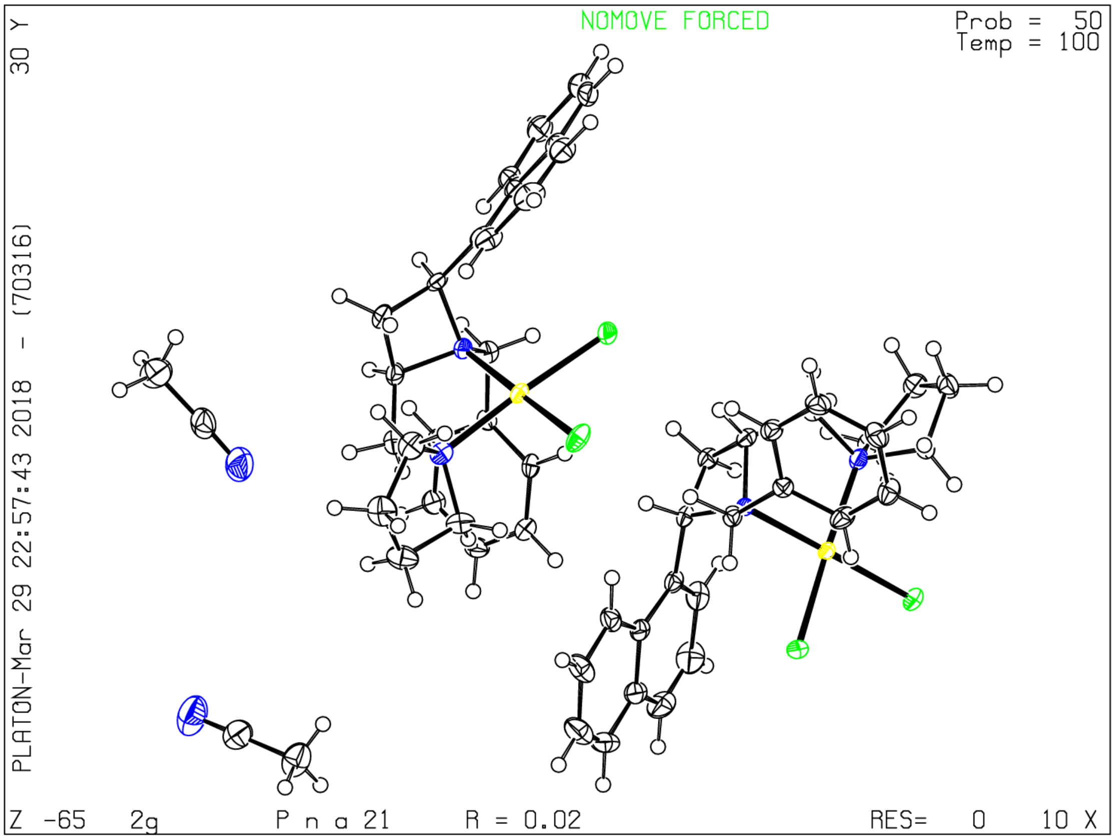

Supplement: Supplementary file 2 [file Data_Sheet_2.ZIP › cif checks/2g_CheckCIF.pdf]
