# Supplementary material for: Palladium and Platinum 2,4-cis-amino Azetidine and Related Complexes
Source: Front Chem. 2018 Jun 21;6:211. doi: 10.3389/fchem.2018.00211 (PMC6021532; doi:10.3389/fchem.2018.00211)

checkCIF/PLATON report

Structure factors have been supplied for datablock(s) 7b

THIS REPORT IS FOR GUIDANCE ONLY. IF USED AS PART OF A REVIEW PROCEDURE FOR PUBLICATION, IT SHOULD NOT REPLACE THE EXPERTISE OF AN EXPERIENCED CRYSTALLOGRAPHIC REFEREE.

No syntax errors found. CIF dictionary Interpreting this report

Datablock: 7b

```
Bond precision:  C-C = 0.0052 A          Wavelength=1.54184

Cell:           a=8.6263(3)       b=12.0854(5)     c=12.8671(7)
                alpha=77.377(4)   beta=75.472(4)   gamma=84.423(3)
Temperature:    100 K

                Calculated                 Reported
Volume          1265.87(10)                1265.87(11)
Space group     P -1                       P -1
Hall group      -P 1                       -P 1
Moiety formula  C27 H34 Cl2 N2 O Pd        C27 H34 Cl2 N2 O Pd
Sum formula     C27 H34 Cl2 N2 O Pd        C27 H34 Cl2 N2 O Pd
Mr              579.86                     579.86
Dx,g cm-3       1.521                      1.521
Z               2                          2
Mu (mm-1)       8.023                      8.023
F000            596.0                      596.0
F000'           598.94
h,k,lmax        10,14,15                   10,14,15
Nref            4816                       4775
Tmin,Tmax       0.476,0.695                0.656,0.878
Tmin'           0.190

Correction method= # Reported T Limits: Tmin=0.656 Tmax=0.878
AbsCorr = GAUSSIAN

Data completeness= 0.991          Theta(max)= 70.074

R(reflections)= 0.0334( 4175)     wR2(reflections)= 0.0890( 4775)

S = 1.029                  Npar= 303
```

The following ALERTS were generated. Each ALERT has the format
test-name_ALERT_alert-type_alert-level.
Click on the hyperlinks for more details of the test.

Alert level C

```
PLAT230_ALERT_2_C Hirshfeld Test Diff for   O1       --C4        .        6.8 s.u.
PLAT911_ALERT_3_C Missing FCF Refl Between Thmin & STh/L=    0.600           4 Report
```

Alert level G

```
PLAT002_ALERT_2_G Number of Distance or Angle Restraints on AtSite           2 Note
PLAT172_ALERT_4_G The CIF-Embedded .res File Contains DFIX Records           1 Report
PLAT793_ALERT_4_G Model has Chirality at N1          (Centro SPGR)           R Verify
PLAT793_ALERT_4_G Model has Chirality at C2          (Centro SPGR)           S Verify
PLAT793_ALERT_4_G Model has Chirality at C4          (Centro SPGR)           S Verify
PLAT860_ALERT_3_G Number of Least-Squares Restraints .............           1 Note
PLAT912_ALERT_4_G Missing # of FCF Reflections Above STh/L=  0.600          38 Note
PLAT978_ALERT_2_G Number C-C Bonds with Positive Residual Density.           1 Info
```

0 **ALERT level A** = Most likely a serious problem - resolve or explain
0 **ALERT level B** = A potentially serious problem, consider carefully
2 **ALERT level C** = Check. Ensure it is not caused by an omission or oversight
8 **ALERT level G** = General information/check it is not something unexpected

0 ALERT type 1 CIF construction/syntax error, inconsistent or missing data
3 ALERT type 2 Indicator that the structure model may be wrong or deficient
2 ALERT type 3 Indicator that the structure quality may be low
5 ALERT type 4 Improvement, methodology, query or suggestion
0 ALERT type 5 Informative message, check

It is advisable to attempt to resolve as many as possible of the alerts in all categories. Often the minor alerts point to easily fixed oversights, errors and omissions in your CIF or refinement strategy, so attention to these fine details can be worthwhile. In order to resolve some of the more serious problems it may be necessary to carry out additional measurements or structure refinements. However, the purpose of your study may justify the reported deviations and the more serious of these should normally be commented upon in the discussion or experimental section of a paper or in the "special_details" fields of the CIF. checkCIF was carefully designed to identify outliers and unusual parameters, but every test has its limitations and alerts that are not important in a particular case may appear. Conversely, the absence of alerts does not guarantee there are no aspects of the results needing attention. It is up to the individual to critically assess their own results and, if necessary, seek expert advice.

Publication of your CIF in IUCr journals

A basic structural check has been run on your CIF. These basic checks will be run on all CIFs submitted for publication in IUCr journals (*Acta Crystallographica*, *Journal of Applied Crystallography*, *Journal of Synchrotron Radiation*); however, if you intend to submit to *Acta Crystallographica Section C* or *E* or *IUCrData*, you should make sure that full publication checks are run on the final version of your CIF prior to submission.

Publication of your CIF in other journals

Please refer to the *Notes for Authors* of the relevant journal for any special instructions relating to CIF submission.

PLATON version of 30/01/2018; check.def file version of 30/01/2018

Datablock 7b - ellipsoid plot

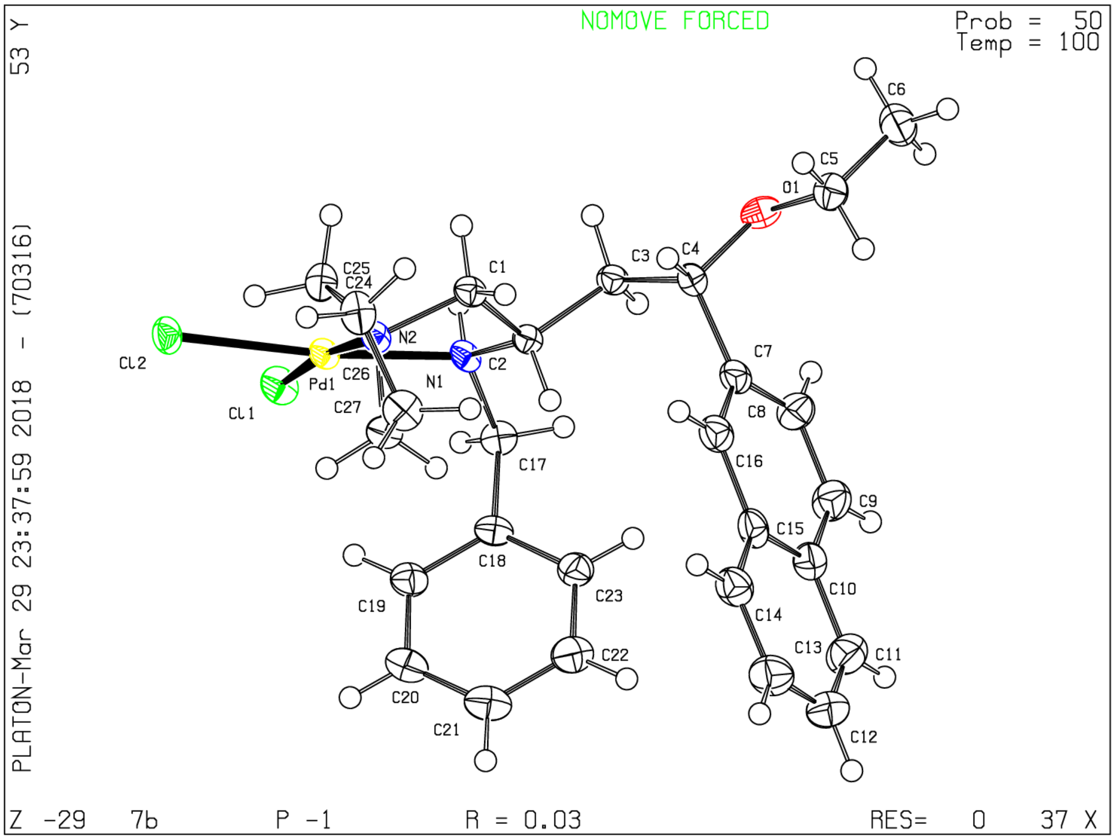

Supplement: Supplementary file 2 [file Data_Sheet_2.ZIP › cif checks/7b_CheckCIF.pdf]
