# Supplementary material for: Palladium and Platinum 2,4-cis-amino Azetidine and Related Complexes
Source: Front Chem. 2018 Jun 21;6:211. doi: 10.3389/fchem.2018.00211 (PMC6021532; doi:10.3389/fchem.2018.00211)

# checkCIF/PLATON report

Structure factors have been supplied for datablock(s) 4a

THIS REPORT IS FOR GUIDANCE ONLY. IF USED AS PART OF A REVIEW PROCEDURE FOR PUBLICATION, IT SHOULD NOT REPLACE THE EXPERTISE OF AN EXPERIENCED CRYSTALLOGRAPHIC REFEREE.

No syntax errors found. CIF dictionary Interpreting this report

## Datablock: 4a

---

```
Bond precision:  C-C = 0.0166 A          Wavelength=0.71073

Cell:           a=10.6958(2)    b=10.4600(2)      c=43.9267(10)
                alpha=90        beta=93.963(1)    gamma=90
Temperature:    120 K

                Calculated                  Reported
Volume          4902.68(17)                 4902.68(17)
Space group     P 21                        P 2(1)
Hall group      P 2yb                       ?
Moiety formula  C24 H26 Cl2 N2 Pt, C2 H3 N C24 H26 Cl2 N2 Pt, C2 H3 N
Sum formula     C26 H29 Cl2 N3 Pt           C26 H29 Cl2 N3 Pt
Mr              649.50                      649.51
Dx,g cm-3       1.760                       1.760
Z               8                           8
Mu (mm-1)       5.961                       5.961
F000            2544.0                      2544.0
F000'           2533.89
h,k,lmax        12,12,52                    12,12,52
Nref            17342[ 9192]                16268
Tmin,Tmax       0.428,0.551                 0.449,0.587
Tmin'           0.381

Correction method= # Reported T Limits: Tmin=0.449 Tmax=0.587
AbsCorr = MULTI-SCAN

Data completeness= 1.77/0.94      Theta(max)= 25.030

R(reflections)= 0.0623( 13783)    wR2(reflections)= 0.0908( 16268)

S = 1.043                   Npar= 1165
```

---

The following ALERTS were generated. Each ALERT has the format
**test-name_ALERT_alert-type_alert-level**.
Click on the hyperlinks for more details of the test.

## Alert level B

```
PLAT910_ALERT_3_B Missing # of FCF Reflection(s) Below Theta(Min).        24 Note
PLAT934_ALERT_3_B Number of (Iobs-Icalc)/SigmaW > 10 Outliers ....         3 Check
```

## Alert level C

```
PLAT029_ALERT_3_C _diffrn_measured_fraction_theta_full value Low .     0.971 Why?
PLAT090_ALERT_3_C Poor Data / Parameter Ratio (Zmax > 18) ........      7.65 Note
PLAT213_ALERT_2_C Atom C217            has ADP max/min Ratio .....       3.2 oblate
PLAT220_ALERT_2_C Non-Solvent  Resd 1  C   Ueq(max)/Ueq(min) Range       3.1 Ratio
PLAT220_ALERT_2_C Non-Solvent  Resd 3  C   Ueq(max)/Ueq(min) Range       3.3 Ratio
PLAT234_ALERT_4_C Large Hirshfeld Difference C107     --C108            0.16 Ang.
PLAT234_ALERT_4_C Large Hirshfeld Difference C120     --C121            0.17 Ang.
PLAT241_ALERT_2_C High   'MainMol' Ueq as Compared to Neighbors of      C217 Check
PLAT342_ALERT_3_C Low Bond Precision on  C-C Bonds ...............   0.01655 Ang.
PLAT906_ALERT_3_C Large K Value in the Analysis of Variance ......     8.011 Check
PLAT906_ALERT_3_C Large K Value in the Analysis of Variance ......     2.534 Check
PLAT911_ALERT_3_C Missing FCF Refl Between Thmin & STh/L=    0.595       247 Report
PLAT972_ALERT_2_C Check Calcd Resid. Dens.  0.95A   From Pt1           -1.80 eA-3
PLAT972_ALERT_2_C Check Calcd Resid. Dens.  0.92A   From Pt1           -1.76 eA-3
PLAT972_ALERT_2_C Check Calcd Resid. Dens.  0.85A   From Pt31          -1.69 eA-3
PLAT972_ALERT_2_C Check Calcd Resid. Dens.  0.89A   From Pt21          -1.59 eA-3
PLAT977_ALERT_2_C Check Negative Difference Density on H12C            -0.39 eA-3
PLAT977_ALERT_2_C Check Negative Difference Density on H50B            -0.42 eA-3
PLAT977_ALERT_2_C Check Negative Difference Density on H209            -0.35 eA-3
PLAT978_ALERT_2_C Number C-C Bonds with Positive Residual Density.         0 Info
```

## Alert level G

```
PLAT002_ALERT_2_G Number of Distance or Angle Restraints on AtSite        20 Note
PLAT003_ALERT_2_G Number of Uiso or Uij Restrained non-H Atoms ...        34 Report
PLAT005_ALERT_5_G No Embedded Refinement Details Found  in the CIF    Please Do !
PLAT033_ALERT_4_G Flack x Value Deviates > 3.0 * sigma from Zero .     0.041 Note
PLAT343_ALERT_2_G Unusual sp3      Angle Range in Main Residue for      C201 Check
PLAT791_ALERT_4_G Model has Chirality at N1          (Chiral SPGR)         S Verify
PLAT791_ALERT_4_G Model has Chirality at N2          (Chiral SPGR)         S Verify
PLAT791_ALERT_4_G Model has Chirality at N101        (Chiral SPGR)         S Verify
PLAT791_ALERT_4_G Model has Chirality at N102        (Chiral SPGR)         S Verify
PLAT791_ALERT_4_G Model has Chirality at N201        (Chiral SPGR)         R Verify
PLAT791_ALERT_4_G Model has Chirality at N202        (Chiral SPGR)         R Verify
PLAT791_ALERT_4_G Model has Chirality at N301        (Chiral SPGR)         R Verify
PLAT791_ALERT_4_G Model has Chirality at N302        (Chiral SPGR)         R Verify
PLAT791_ALERT_4_G Model has Chirality at C1          (Chiral SPGR)         R Verify
PLAT791_ALERT_4_G Model has Chirality at C3          (Chiral SPGR)         S Verify
PLAT791_ALERT_4_G Model has Chirality at C101        (Chiral SPGR)         R Verify
PLAT791_ALERT_4_G Model has Chirality at C103        (Chiral SPGR)         S Verify
PLAT791_ALERT_4_G Model has Chirality at C201        (Chiral SPGR)         S Verify
PLAT791_ALERT_4_G Model has Chirality at C203        (Chiral SPGR)         R Verify
PLAT791_ALERT_4_G Model has Chirality at C301        (Chiral SPGR)         S Verify
PLAT791_ALERT_4_G Model has Chirality at C303        (Chiral SPGR)         R Verify
PLAT860_ALERT_3_G Number of Least-Squares Restraints .............       277 Note
PLAT899_ALERT_4_G SHELXL97   is Deprecated and Succeeded by SHELXL      2018 Note
PLAT909_ALERT_3_G Percentage of I>2sig(I) Data at Theta(Max) Still       66% Note
PLAT913_ALERT_3_G Missing # of Very Strong Reflections in FCF ....         1 Note
```

0 **ALERT level A** = Most likely a serious problem - resolve or explain
2 **ALERT level B** = A potentially serious problem, consider carefully
20 **ALERT level C** = Check. Ensure it is not caused by an omission or oversight
25 **ALERT level G** = General information/check it is not something unexpected

```
 0 ALERT type 1 CIF construction/syntax error, inconsistent or missing data
15 ALERT type 2 Indicator that the structure model may be wrong or deficient
11 ALERT type 3 Indicator that the structure quality may be low
20 ALERT type 4 Improvement, methodology, query or suggestion
 1 ALERT type 5 Informative message, check
```

---

It is advisable to attempt to resolve as many as possible of the alerts in all categories. Often the minor alerts point to easily fixed oversights, errors and omissions in your CIF or refinement strategy, so attention to these fine details can be worthwhile. In order to resolve some of the more serious problems it may be necessary to carry out additional measurements or structure refinements. However, the purpose of your study may justify the reported deviations and the more serious of these should normally be commented upon in the discussion or experimental section of a paper or in the "special_details" fields of the CIF. checkCIF was carefully designed to identify outliers and unusual parameters, but every test has its limitations and alerts that are not important in a particular case may appear. Conversely, the absence of alerts does not guarantee there are no aspects of the results needing attention. It is up to the individual to critically assess their own results and, if necessary, seek expert advice.

**Publication of your CIF in IUCr journals**

A basic structural check has been run on your CIF. These basic checks will be run on all CIFs submitted for publication in IUCr journals (*Acta Crystallographica*, *Journal of Applied Crystallography*, *Journal of Synchrotron Radiation*); however, if you intend to submit to *Acta Crystallographica Section C* or *E* or *IUCrData*, you should make sure that full publication checks are run on the final version of your CIF prior to submission.

**Publication of your CIF in other journals**

Please refer to the *Notes for Authors* of the relevant journal for any special instructions relating to CIF submission.

---

**PLATON version of 30/01/2018; check.def file version of 30/01/2018**

**Datablock 4a** - ellipsoid plot

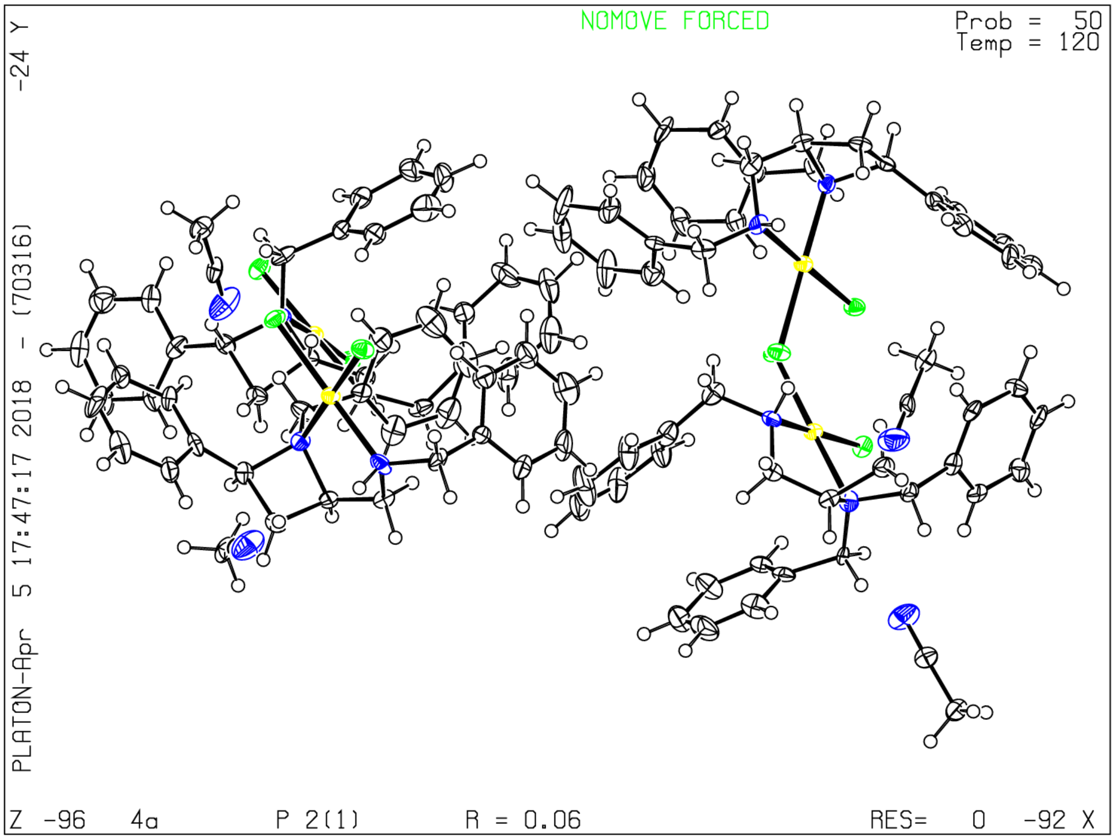

Supplement: Supplementary file 2 [file Data_Sheet_2.ZIP › cif checks/4a_CheckCIF.pdf]
