# Supplementary material for: Palladium and Platinum 2,4-cis-amino Azetidine and Related Complexes
Source: Front Chem. 2018 Jun 21;6:211. doi: 10.3389/fchem.2018.00211 (PMC6021532; doi:10.3389/fchem.2018.00211)

checkCIF/PLATON report

Structure factors have been supplied for datablock(s) 2a

THIS REPORT IS FOR GUIDANCE ONLY. IF USED AS PART OF A REVIEW PROCEDURE FOR PUBLICATION, IT SHOULD NOT REPLACE THE EXPERTISE OF AN EXPERIENCED CRYSTALLOGRAPHIC REFEREE.

No syntax errors found. CIF dictionary Interpreting this report

Datablock: 2a

```
Bond precision:  C-C = 0.0187 A          Wavelength=1.54184

Cell:            a=10.6927(1)     b=10.4108(1)       c=43.8600(4)
                 alpha=90         beta=93.886(1)     gamma=90
Temperature:     100 K
```

	Calculated	Reported
Volume	4871.25(8)	4871.25(8)
Space group	P 21	P 1 21 1
Hall group	P 2yb	P 2yb
Moiety formula	C24 H26 Cl2 N2 Pd, C2 H3 N	C24 H26 Cl2 N2 Pd, C2 H3 N
Sum formula	C26 H29 Cl2 N3 Pd	C26 H29 Cl2 N3 Pd
Mr	560.82	560.82
Dx,g cm-3	1.529	1.529
Z	8	8
Mu (mm-1)	8.303	8.303
F000	2288.0	2288.0
F000’	2299.45	
h,k,lmax	12,12,52	12,12,52
Nref	17240[9141]	17235
Tmin,Tmax	0.443,0.720	0.687,0.903
Tmin’	0.186	

```
Correction method= # Reported T Limits: Tmin=0.687 Tmax=0.903
AbsCorr = GAUSSIAN

Data completeness= 1.89/1.00      Theta(max)= 66.599

R(reflections)= 0.0473( 16784)    wR2(reflections)= 0.1155( 17235)

S = 1.122                  Npar= 1115
```

The following ALERTS were generated. Each ALERT has the format
test-name_ALERT_alert-type_alert-level.
Click on the hyperlinks for more details of the test.

Alert level C

```
PLAT018_ALERT_1_C _diffrn_measured_fraction_theta_max .NE. *_full          ! Check
```

Author Response: _diffrn_reflns_theta_max is reported as 66.599 degrees while _diffrn_reflns_theta_full is reported as 67.684 degrees. As these are not equal there is no reason for _diffrn_measured_fraction_theta_max to be reported as equal to _diffrn_measured_fraction_theta_full.

```
PLAT094_ALERT_2_C Ratio of Maximum / Minimum Residual Density ....       2.11 Report
PLAT213_ALERT_2_C Atom N2              has ADP max/min Ratio .....        3.6 oblate
PLAT213_ALERT_2_C Atom C2              has ADP max/min Ratio .....        3.3 prolat
PLAT220_ALERT_2_C Non-Solvent  Resd 2  C   Ueq(max)/Ueq(min) Range        3.7 Ratio
PLAT342_ALERT_3_C Low Bond Precision on  C-C Bonds ...............    0.01867 Ang.
PLAT971_ALERT_2_C Check Calcd Resid. Dens.  0.85A   From Pd31            2.47 eA-3
PLAT971_ALERT_2_C Check Calcd Resid. Dens.  1.01A   From Pd21            2.30 eA-3
PLAT971_ALERT_2_C Check Calcd Resid. Dens.  0.95A   From Pd1             2.29 eA-3
PLAT971_ALERT_2_C Check Calcd Resid. Dens.  1.14A   From Pd1             2.27 eA-3
PLAT971_ALERT_2_C Check Calcd Resid. Dens.  0.88A   From Pd11            2.21 eA-3
PLAT971_ALERT_2_C Check Calcd Resid. Dens.  0.90A   From Pd11            2.04 eA-3
PLAT971_ALERT_2_C Check Calcd Resid. Dens.  0.96A   From Pd31            2.03 eA-3
PLAT971_ALERT_2_C Check Calcd Resid. Dens.  1.20A   From Pd21            1.97 eA-3
PLAT978_ALERT_2_C Number C-C Bonds with Positive Residual Density.          0 Info
```

Alert level G

```
PLAT002_ALERT_2_G Number of Distance or Angle Restraints on AtSite          4 Note
PLAT083_ALERT_2_G SHELXL Second Parameter in WGHT  Unusually Large      50.79 Why ?
PLAT142_ALERT_4_G s.u. on b - Axis Small or Missing ..............    0.00010 Ang.
PLAT143_ALERT_4_G s.u. on c - Axis Small or Missing ..............    0.00040 Ang.
PLAT171_ALERT_4_G The CIF-Embedded .res File Contains EADP Records          9 Report
PLAT172_ALERT_4_G The CIF-Embedded .res File Contains DFIX Records          1 Report
PLAT791_ALERT_4_G Model has Chirality at N1          (Chiral SPGR)          R Verify
PLAT791_ALERT_4_G Model has Chirality at N2          (Chiral SPGR)          R Verify
PLAT791_ALERT_4_G Model has Chirality at N101        (Chiral SPGR)          S Verify
PLAT791_ALERT_4_G Model has Chirality at N102        (Chiral SPGR)          S Verify
PLAT791_ALERT_4_G Model has Chirality at N201        (Chiral SPGR)          R Verify
PLAT791_ALERT_4_G Model has Chirality at N202        (Chiral SPGR)          R Verify
PLAT791_ALERT_4_G Model has Chirality at N301        (Chiral SPGR)          S Verify
PLAT791_ALERT_4_G Model has Chirality at N302        (Chiral SPGR)          S Verify
PLAT791_ALERT_4_G Model has Chirality at C1          (Chiral SPGR)          S Verify
PLAT791_ALERT_4_G Model has Chirality at C3          (Chiral SPGR)          R Verify
PLAT791_ALERT_4_G Model has Chirality at C101        (Chiral SPGR)          R Verify
PLAT791_ALERT_4_G Model has Chirality at C103        (Chiral SPGR)          S Verify
PLAT791_ALERT_4_G Model has Chirality at C201        (Chiral SPGR)          S Verify
PLAT791_ALERT_4_G Model has Chirality at C203        (Chiral SPGR)          R Verify
PLAT791_ALERT_4_G Model has Chirality at C301        (Chiral SPGR)          R Verify
PLAT791_ALERT_4_G Model has Chirality at C303        (Chiral SPGR)          S Verify
PLAT860_ALERT_3_G Number of Least-Squares Restraints .............          3 Note
PLAT909_ALERT_3_G Percentage of I>2sig(I) Data at Theta(Max) Still        95% Note
PLAT910_ALERT_3_G Missing # of FCF Reflection(s) Below Theta(Min).          2 Note
PLAT933_ALERT_2_G Number of OMIT Records in Embedded .res File ...          4 Note
```

0 **ALERT level A** = Most likely a serious problem - resolve or explain

```
  0 ALERT level B = A potentially serious problem, consider carefully
 15 ALERT level C = Check. Ensure it is not caused by an omission or oversight
 26 ALERT level G = General information/check it is not something unexpected

  1 ALERT type 1 CIF construction/syntax error, inconsistent or missing data
 16 ALERT type 2 Indicator that the structure model may be wrong or deficient
  4 ALERT type 3 Indicator that the structure quality may be low
 20 ALERT type 4 Improvement, methodology, query or suggestion
  0 ALERT type 5 Informative message, check
```

Validation response form

Please find below a validation response form (VRF) that can be filled in and pasted into your CIF.

```
# start Validation Reply Form
_vrf_PLAT094_2a
;
PROBLEM: Ratio of Maximum / Minimum Residual Density ....       2.11 Report
RESPONSE: ...
;
_vrf_PLAT213_2a
;
PROBLEM: Atom N2              has ADP max/min Ratio .....        3.6 oblate
RESPONSE: ...
;
_vrf_PLAT220_2a
;
PROBLEM: Non-Solvent  Resd 2  C   Ueq(max)/Ueq(min) Range        3.7 Ratio
RESPONSE: ...
;
_vrf_PLAT342_2a
;
PROBLEM: Low Bond Precision on  C-C Bonds ..............    0.01867 Ang.
RESPONSE: ...
;
_vrf_PLAT971_2a
;
PROBLEM: Check Calcd Resid. Dens.  0.85A   From Pd31            2.47 eA-3
RESPONSE: ...
;
_vrf_PLAT978_2a
;
PROBLEM: Number C-C Bonds with Positive Residual Density.          0 Info
RESPONSE: ...
;
# end Validation Reply Form
```

It is advisable to attempt to resolve as many as possible of the alerts in all categories. Often the minor alerts point to easily fixed oversights, errors and omissions in your CIF or refinement strategy, so attention to these fine details can be worthwhile. In order to resolve some of the more serious problems it may be necessary to carry out additional measurements or structure refinements. However, the purpose of your study may justify the reported deviations and the more serious of these should normally be commented upon in the discussion or experimental section of a paper or in the "special_details" fields of the CIF. checkCIF was carefully designed to identify outliers and unusual parameters, but every test has its limitations and alerts that are not important in a particular case may appear. Conversely, the absence of alerts does not guarantee there are no aspects of the results needing attention. It is up to the individual to critically assess their own results and, if necessary, seek expert advice.

Publication of your CIF in IUCr journals

A basic structural check has been run on your CIF. These basic checks will be run on all CIFs submitted for publication in IUCr journals (*Acta Crystallographica*, *Journal of Applied Crystallography*, *Journal of Synchrotron Radiation*); however, if you intend to submit to *Acta Crystallographica Section C* or *E* or *IUCrData*, you should make sure that full publication checks are run on the final version of your CIF prior to submission.

Publication of your CIF in other journals

Please refer to the *Notes for Authors* of the relevant journal for any special instructions relating to CIF submission.

PLATON version of 30/01/2018; check.def file version of 30/01/2018

Datablock 2a - ellipsoid plot

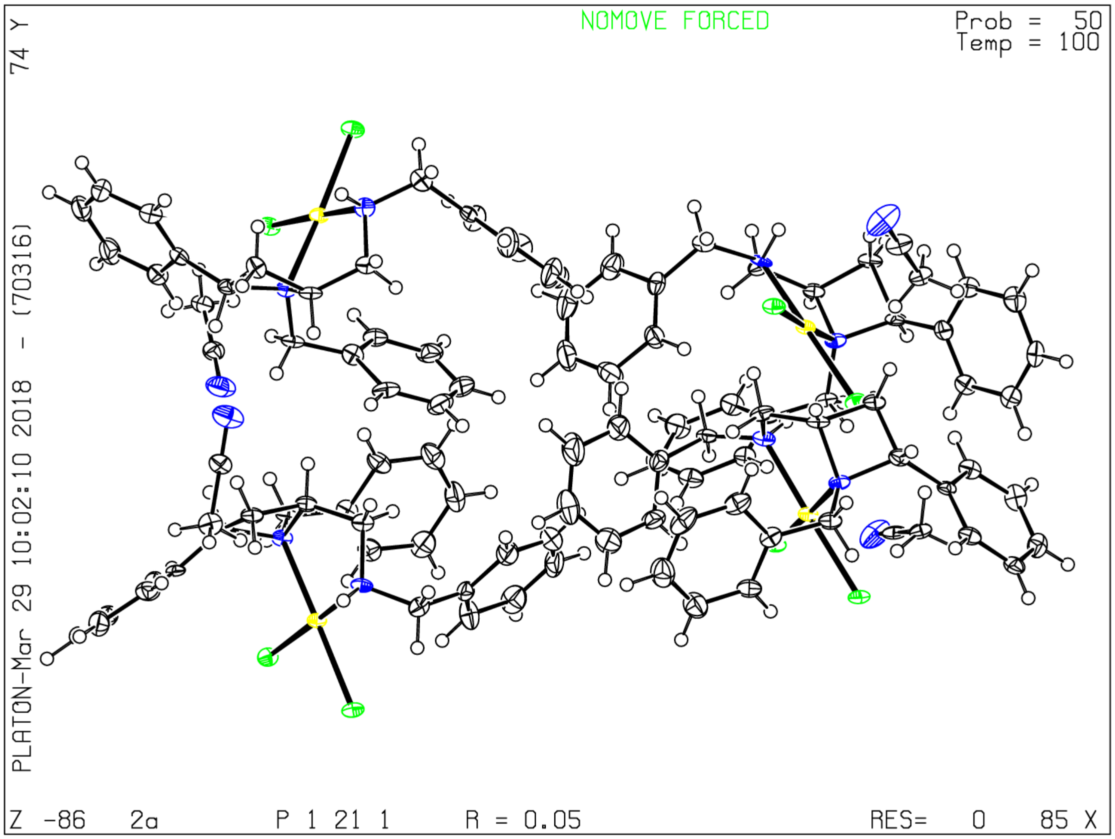

Supplement: Supplementary file 2 [file 2a_CheckCIF.pdf › cif checks/2a_CheckCIF.pdf]
