# Supplementary material for: Palladium and Platinum 2,4-cis-amino Azetidine and Related Complexes
Source: Front Chem. 2018 Jun 21;6:211. doi: 10.3389/fchem.2018.00211 (PMC6021532; doi:10.3389/fchem.2018.00211)

checkCIF/PLATON report

Structure factors have been supplied for datablock(s) 2b

THIS REPORT IS FOR GUIDANCE ONLY. IF USED AS PART OF A REVIEW PROCEDURE FOR PUBLICATION, IT SHOULD NOT REPLACE THE EXPERTISE OF AN EXPERIENCED CRYSTALLOGRAPHIC REFEREE.

No syntax errors found. CIF dictionary Interpreting this report

Datablock: 2b

Bond precision: C-C = 0.0060 A Wavelength=0.71073

Cell:	a=13.4719(2)	b=19.3528(2)	c=21.6914(3)
	alpha=94.412(1)	beta=106.425(1)	gamma=108.667(1)
Temperature:	120 K		

	Calculated	Reported
Volume	5051.23(13)	5051.22(12)
Space group	P -1	P -1
Hall group	-P 1	-P 1
Moiety formula	C25 H28 Cl2 N2 O Pd, 0.1(C4 H10 O)	C25 H28 Cl2 N2 O Pd, 0.1(C4 H10 O)
Sum formula	C25.40 H29 Cl2 N2 O1.10 Pd	C25.40 H29 Cl2 N2 O1.10 Pd
Mr	557.21	557.20
Dx,g cm-3	1.465	1.465
Z	8	8
Mu (mm-1)	0.966	0.966
F000	2273.6	2274.0
F000’	2268.62	
h,k,lmax	17,25,28	17,25,28
Nref	23331	23199
Tmin,Tmax	0.870,0.935	0.801,0.935
Tmin’	0.793	

Correction method= # Reported T Limits: Tmin=0.801 Tmax=0.935
AbsCorr = MULTI-SCAN

Data completeness= 0.994 Theta(max)= 27.549

R(reflections)= 0.0377(19395) wR2(reflections)= 0.0910(23199)

S = 1.035 Npar= 1155

The following ALERTS were generated. Each ALERT has the format
test-name_ALERT_alert-type_alert-level.
Click on the hyperlinks for more details of the test.

Alert level B

```
PLAT910_ALERT_3_B Missing # of FCF Reflection(s) Below Theta(Min).         31 Note
```

Author Response: These data were collected by the UK National Crystallography Service in 2010 and at that time this CheckCIF Alert did not appear. It is no longer possible to try to correct this issue.

Alert level C

```
PLAT077_ALERT_4_C Unitcell Contains Non-integer Number of Atoms ..     Please Check
PLAT220_ALERT_2_C Non-Solvent  Resd 2  C   Ueq(max)/Ueq(min) Range        3.9 Ratio
PLAT220_ALERT_2_C Non-Solvent  Resd 4  C   Ueq(max)/Ueq(min) Range        3.1 Ratio
PLAT222_ALERT_3_C Non-Solv.  Resd 2  H   Uiso(max)/Uiso(min) Range        4.3 Ratio
PLAT241_ALERT_2_C High   'MainMol' Ueq as Compared to Neighbors of       C108 Check
PLAT241_ALERT_2_C High   'MainMol' Ueq as Compared to Neighbors of       C122 Check
PLAT250_ALERT_2_C Large U3/U1 Ratio for Average U(i,j) Tensor ....        2.6 Note
PLAT411_ALERT_2_C Short Inter H...H Contact  H5B      ..H103             2.12 Ang.
PLAT411_ALERT_2_C Short Inter H...H Contact  H12A     ..H25              2.13 Ang.
PLAT601_ALERT_2_C Structure Contains Solvent Accessible VOIDS of .         34 Ang**3
PLAT906_ALERT_3_C Large K Value in the Analysis of Variance ......      3.099 Check
PLAT911_ALERT_3_C Missing FCF Refl Between Thmin & STh/L=    0.600         13 Report
```

Alert level G

```
PLAT002_ALERT_2_G Number of Distance or Angle Restraints on AtSite         14 Note
PLAT003_ALERT_2_G Number of Uiso or Uij Restrained non-H Atoms ...          6 Report
PLAT066_ALERT_1_G Predicted and Reported Tmin&Tmax Range Identical          ? Check
PLAT068_ALERT_1_G Reported F000 Differs from Calcd (or Missing)...     Please Check
PLAT083_ALERT_2_G SHELXL Second Parameter in WGHT  Unusually Large       8.54 Why ?
PLAT154_ALERT_1_G The s.u.'s on the Cell Angles are Equal ..(Note)      0.001 Degree
PLAT172_ALERT_4_G The CIF-Embedded .res File Contains DFIX Records          2 Report
PLAT173_ALERT_4_G The CIF-Embedded .res File Contains DANG Records          1 Report
PLAT177_ALERT_4_G The CIF-Embedded .res File Contains DELU Records          1 Report
PLAT178_ALERT_4_G The CIF-Embedded .res File Contains SIMU Records          1 Report
PLAT300_ALERT_4_G Atom Site Occupancy of O401      Constrained at         0.4 Check
PLAT300_ALERT_4_G Atom Site Occupancy of C401      Constrained at         0.4 Check
PLAT300_ALERT_4_G Atom Site Occupancy of C402      Constrained at         0.4 Check
PLAT300_ALERT_4_G Atom Site Occupancy of C403      Constrained at         0.4 Check
PLAT300_ALERT_4_G Atom Site Occupancy of C404      Constrained at         0.4 Check
PLAT300_ALERT_4_G Atom Site Occupancy of H40A      Constrained at         0.4 Check
PLAT300_ALERT_4_G Atom Site Occupancy of H40B      Constrained at         0.4 Check
PLAT300_ALERT_4_G Atom Site Occupancy of H40C      Constrained at         0.4 Check
PLAT300_ALERT_4_G Atom Site Occupancy of H40D      Constrained at         0.4 Check
PLAT300_ALERT_4_G Atom Site Occupancy of H40E      Constrained at         0.4 Check
PLAT300_ALERT_4_G Atom Site Occupancy of H40F      Constrained at         0.4 Check
PLAT300_ALERT_4_G Atom Site Occupancy of H40G      Constrained at         0.4 Check
PLAT300_ALERT_4_G Atom Site Occupancy of H40H      Constrained at         0.4 Check
PLAT300_ALERT_4_G Atom Site Occupancy of H40I      Constrained at         0.4 Check
PLAT300_ALERT_4_G Atom Site Occupancy of H40J      Constrained at         0.4 Check
PLAT302_ALERT_4_G Anion/Solvent/Minor-Residue Disorder (Resd  5  )       100% Note
PLAT793_ALERT_4_G Model has Chirality at N1         (Centro SPGR)           S Verify
PLAT793_ALERT_4_G Model has Chirality at N2         (Centro SPGR)           S Verify
PLAT793_ALERT_4_G Model has Chirality at N101       (Centro SPGR)           S Verify
```

```
PLAT793_ALERT_4_G Model has Chirality at N102        (Centro SPGR)          S Verify
PLAT793_ALERT_4_G Model has Chirality at N201        (Centro SPGR)          S Verify
PLAT793_ALERT_4_G Model has Chirality at N202        (Centro SPGR)          S Verify
PLAT793_ALERT_4_G Model has Chirality at N301        (Centro SPGR)          S Verify
PLAT793_ALERT_4_G Model has Chirality at N302        (Centro SPGR)          S Verify
PLAT793_ALERT_4_G Model has Chirality at C1          (Centro SPGR)          R Verify
PLAT793_ALERT_4_G Model has Chirality at C3          (Centro SPGR)          S Verify
PLAT793_ALERT_4_G Model has Chirality at C101        (Centro SPGR)          R Verify
PLAT793_ALERT_4_G Model has Chirality at C103        (Centro SPGR)          S Verify
PLAT793_ALERT_4_G Model has Chirality at C201        (Centro SPGR)          R Verify
PLAT793_ALERT_4_G Model has Chirality at C203        (Centro SPGR)          S Verify
PLAT793_ALERT_4_G Model has Chirality at C301        (Centro SPGR)          R Verify
PLAT793_ALERT_4_G Model has Chirality at C303        (Centro SPGR)          S Verify
PLAT860_ALERT_3_G Number of Least-Squares Restraints .............         57 Note
PLAT912_ALERT_4_G Missing # of FCF Reflections Above STh/L=  0.600         87 Note
PLAT913_ALERT_3_G Missing # of Very Strong Reflections in FCF ....          1 Note
PLAT933_ALERT_2_G Number of OMIT Records in Embedded .res File ...          9 Note
PLAT978_ALERT_2_G Number C-C Bonds with Positive Residual Density.          1 Info
```

---

0 **ALERT level A** = Most likely a serious problem - resolve or explain
1 **ALERT level B** = A potentially serious problem, consider carefully
12 **ALERT level C** = Check. Ensure it is not caused by an omission or oversight
47 **ALERT level G** = General information/check it is not something unexpected

3 ALERT type 1 CIF construction/syntax error, inconsistent or missing data
13 ALERT type 2 Indicator that the structure model may be wrong or deficient
6 ALERT type 3 Indicator that the structure quality may be low
38 ALERT type 4 Improvement, methodology, query or suggestion
0 ALERT type 5 Informative message, check

It is advisable to attempt to resolve as many as possible of the alerts in all categories. Often the minor alerts point to easily fixed oversights, errors and omissions in your CIF or refinement strategy, so attention to these fine details can be worthwhile. In order to resolve some of the more serious problems it may be necessary to carry out additional measurements or structure refinements. However, the purpose of your study may justify the reported deviations and the more serious of these should normally be commented upon in the discussion or experimental section of a paper or in the "special_details" fields of the CIF. checkCIF was carefully designed to identify outliers and unusual parameters, but every test has its limitations and alerts that are not important in a particular case may appear. Conversely, the absence of alerts does not guarantee there are no aspects of the results needing attention. It is up to the individual to critically assess their own results and, if necessary, seek expert advice.

Publication of your CIF in IUCr journals

A basic structural check has been run on your CIF. These basic checks will be run on all CIFs submitted for publication in IUCr journals (*Acta Crystallographica*, *Journal of Applied Crystallography*, *Journal of Synchrotron Radiation*); however, if you intend to submit to *Acta Crystallographica Section C* or *E* or *IUCrData*, you should make sure that full publication checks are run on the final version of your CIF prior to submission.

Publication of your CIF in other journals

Please refer to the *Notes for Authors* of the relevant journal for any special instructions relating to CIF submission.

PLATON version of 30/01/2018; check.def file version of 30/01/2018

Datablock 2b - ellipsoid plot

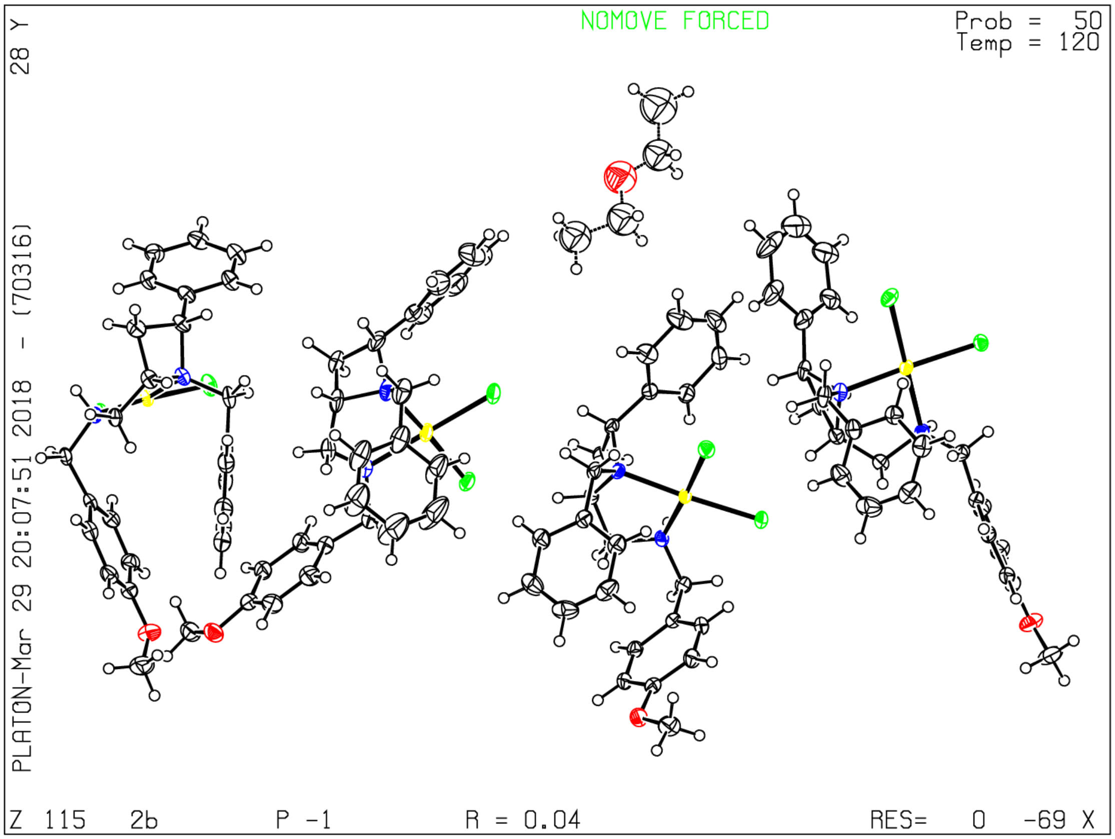

Supplement: Supplementary file 2 [file 2b_CheckCIF.pdf › cif checks/2b_CheckCIF.pdf]
